# Artificial Intelligence for Opioid Safety Surveillance from Clinical Text: A Clinically Focused Review

**DOI:** 10.3390/jcm15041649

**Published:** 2026-02-22

**Authors:** Md Muntasir Zitu, Dwight Owen, Ashish Manne, Yuxi Zhu, Samar Binkheder, Lang Li

**Affiliations:** 1 Department of Machine Learning, Moffitt Cancer Center and Research Institute, Tampa, FL 33612, USA; 2 Department of Medicine, College of Medicine, The Ohio State University, Columbus, OH 43210, USA; 3 Department of Pediatrics, University Hospitals Rainbow Babies & Children’s Hospital, Cleveland, OH 44106, USA; 4 Medical Informatics and E-Learning Unit, Medical Education Department, College of Medicine, King Saud University, Riyadh 12372, Saudi Arabia; 5 Department of Biomedical Informatics, College of Medicine, The Ohio State University, Columbus, OH 43210, USA

**Keywords:** opioid safety, opioid use disorder, pharmacovigilance, clinical text, artificial intelligence, natural language processing, large language models, generative artificial intelligence (GenAI), electronic health records

## Abstract

Opioid-related iatrogenic harms, including opioid use disorder, overdose, and opioid-induced respiratory depression, constitute a major patient safety challenge. Although clinicians document key safety signals in unstructured clinical narratives, many of these indicators are not readily captured by conventional surveillance approaches that rely on structured administrative data. This clinically focused narrative review synthesizes 47 empirical studies published between 2009 and 2025 that applied artificial intelligence (AI) methods to identify opioid-related harms from clinical text and to address the resulting ascertainment gap. Across studies, administrative coding systems, including ICD-10, often under-ascertain opioid-related events, whereas text-based AI can identify additional cases and contextual details often documented primarily in narrative records, such as fluctuating mental status, suspected drug causality, and responses to naloxone. Methodologically, the literature has progressed from interpretable rule-based lexicons to machine learning and deep learning models and, more recently, to transformer-based approaches, including large language models (LLMs) for classification and schema-driven extraction. Rule-based systems established the feasibility of transparent surveillance and frequently recovered clinically documented cases missed by billing codes. Subsequent supervised and deep learning approaches expanded scalability and, in a smaller subset of studies, were integrated into electronic health record workflows with operational metrics reported. More recent transformer- and LLM-based studies emphasize richer extraction schemas and benchmark development, including characterization of overdose context and intentionality and identification of potential prodromal neurocognitive signals, although external validation, calibration, and prospective outcome evaluation remain inconsistently reported. Given that the evidence base is predominantly retrospective and that clinical workflow studies remain comparatively few, a pragmatic near-term clinical role is to provide detection-to-triage decision support rather than autonomous diagnosis, in which systems surface candidate cases with reviewable evidence for clinician adjudication. Future progress will require greater standardization of phenotype definitions, routine equity auditing and subgroup reporting, stronger external validation and calibration at operational thresholds, and a shift from retrospective discrimination metrics toward prospective assessments of the clinical workflow impact, clinical utility, and patient-centered outcomes.

## 1. Introduction

Opioids represent a double-edged therapeutic cornerstone, essential for managing acute and chronic pain across the continuum of care, spanning from the oncology suite and perioperative settings to emergency departments and outpatient clinics [1,2,3]. However, this therapeutic utility is accompanied by a spectrum of preventable iatrogenic harms, including accidental overdose, the development of opioid use disorder (OUD), and the life-threatening risk of opioid-induced respiratory depression (OIRD) [3,4,5,6]. The scale of this burden is substantial. For example, in 2023, opioid-involved overdoses claimed 79,358 lives in the United States alone, accounting for ~76% of all drug overdose deaths [7,8,9,10].

**Figure 1:**
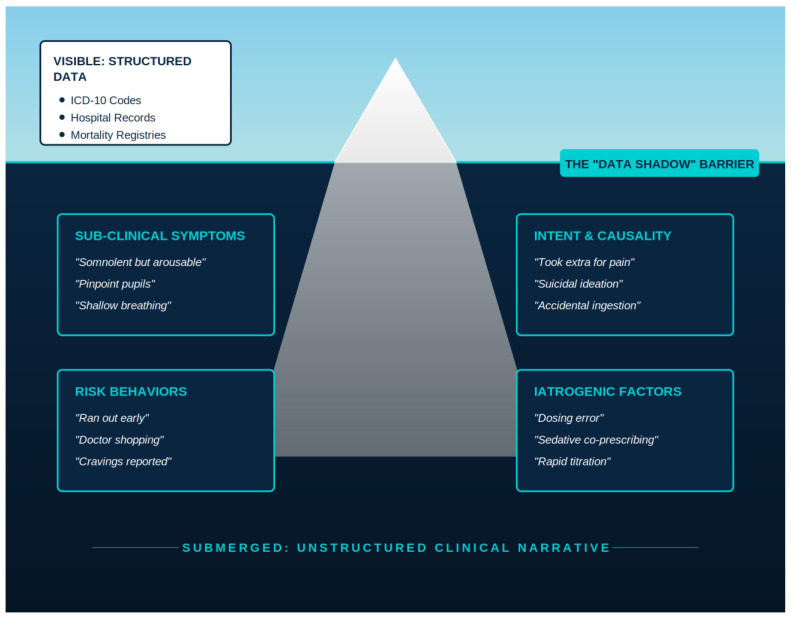
Visualization of the broader gap concept that motivates the methodological shift to a natural language processing (NLP)-based AI approach. While structured EHR fields capture confirmed diagnoses, the majority of actionable safety signals, including subtle symptoms and behavioral precursors, remain embedded within unstructured clinical narratives.

Critically, subtle early indicators of these adverse events, such as fluctuating mental status, suspected causality, or a nuanced response to naloxone, are often documented primarily within unstructured clinical narratives [11,12,13,14]. Because traditional surveillance systems rely on structured data, these narrative signals remain effectively invisible, a situation exacerbated by their dispersion across heterogeneous note types, including Emergency Medical Services (EMS) narratives, nursing progress notes, and discharge summaries [15,16,17]. This systemic omission contributes to a pervasive under-ascertainment of harm, as administrative billing codes and pharmacy records lack the granularity to capture clinical context or causal attribution. Previous studies indicate that International Classification of Diseases (ICD)-based algorithms substantially undercount opioid-related adverse events. For example, a recent validation study found that ICD-10 codes detected only 56% of expert-confirmed opioid misuse cases in the emergency department, with sensitivity dropping to 31% among admitted patients versus 65% among those discharged [18,19,20]. The resulting gap between clinical documentation and administrative representation across key safety domains is detailed in Table 1 and conceptualized in Figure 1, motivating clinical-text-based approaches that extend case finding beyond administrative claims and ICD codes.

The clinical challenge of interpreting these narrative signals is further compounded by sociotechnical complexities. Opioid-related documentation is inherently context-dependent. For example, a mention of “respiratory failure” may represent an active iatrogenic event, a historical risk factor, or merely a differential diagnosis under consideration [21,22,23]. Furthermore, the ethical stakes of automated surveillance are uniquely high. While false negatives represent missed opportunities for life-saving harm reduction, false positives carry the risk of patient stigmatization or the inadvertent restriction of appropriate analgesia for patients in genuine pain [1,24]. This concern is not theoretical; a simulated patient study found that 40.7% of primary care clinics would not accept new patients already receiving long-term opioids, suggesting that surveillance-driven labeling may create barriers to care [25,26,27]. Consequently, evaluating AI models in this space must extend beyond traditional accuracy metrics to focus on clinical interpretability, workflow integration, and governance [26,27,28].

Methodologically, the field has evolved from early artificial intelligence (AI) approaches, broadly defined as computer systems designed to perform tasks that typically require human intelligence, toward natural language processing (NLP) methods that enable computers to analyze and interpret clinical text, often using rule-based lexicons such as curated term lists and pattern matching rules [29,30]. The field subsequently progressed to machine learning (ML) models, which learn predictive patterns from data rather than relying on hand-coded rules, and deep learning (DL) models, a subset of ML that uses multi-layer neural networks to learn complex representations from electronic health record (EHR) data, including structured fields and free text [29,30]. More recently, transformer-based neural architectures designed to model contextual relationships across long sequences have enabled large language models (LLMs), which are large-scale transformer models trained on extensive text corpora and adapted for clinical tasks such as schema-driven extraction and synthesis of clinically relevant information [31,32,33]. However, each methodological phase presents distinct trade-offs in transparency, scalability, and model drift management within safety-critical settings.

Current literature suggests that while fully autonomous detection is the long-term goal, the most viable current strategy is detection-to-triage. In this workflow, the AI acts as a high-sensitivity filter, surfacing candidate cases and specific “note spans” for clinician adjudication [32,33]. This approach prioritizes human oversight and appropriate routing, though the optimal operational model for hospital-wide integration remains a key subject for prospective evaluation.

### Study Aim and Goals

Against this backdrop, this clinically focused narrative review synthesizes empirical studies published from 2009 through December 2025 that leverage clinical texts to identify opioid-related safety signals and harms. Our review aims to:

(a) Map the clinical targets addressed in the literature, documenting how AI identifies a cohort of patients missed by administrative data.
(b) Summarize the technical evolution of models while critically evaluating the limitations of current reference standards and validation designs.
(c) Translate technical findings into clinical meaning. Clarify the practical clinical role for these tools, distinguishing between detect-to-triage support for human review and longitudinal safety surveillance, while identifying risks such as bias and classification error that limit autonomous deployment without human oversight.
(d) Synthesize a roadmap for clinical transition, shifting emphasis from retrospective detection accuracy toward prospective assessments of clinical utility.

By organizing the evidence around clinical objectives and deployment-relevant considerations, this review seeks to provide clinicians and informatics leaders with a clear understanding of what the literature supports today and what must still be demonstrated before clinical-text-based opioid safety tools can be reliably integrated into routine clinical workflows. Supplementary Table S1 (Abbreviations and Acronyms) and Supplementary Table S2 (Key Technical Concepts and Workflow Terms) are intended to assist readers in navigating the manuscript and are recommended for review to facilitate a clearer understanding of the content.

## 2. Methods

### 2.1. Scope of Review (Inclusion and Exclusion)

**Figure 2:**
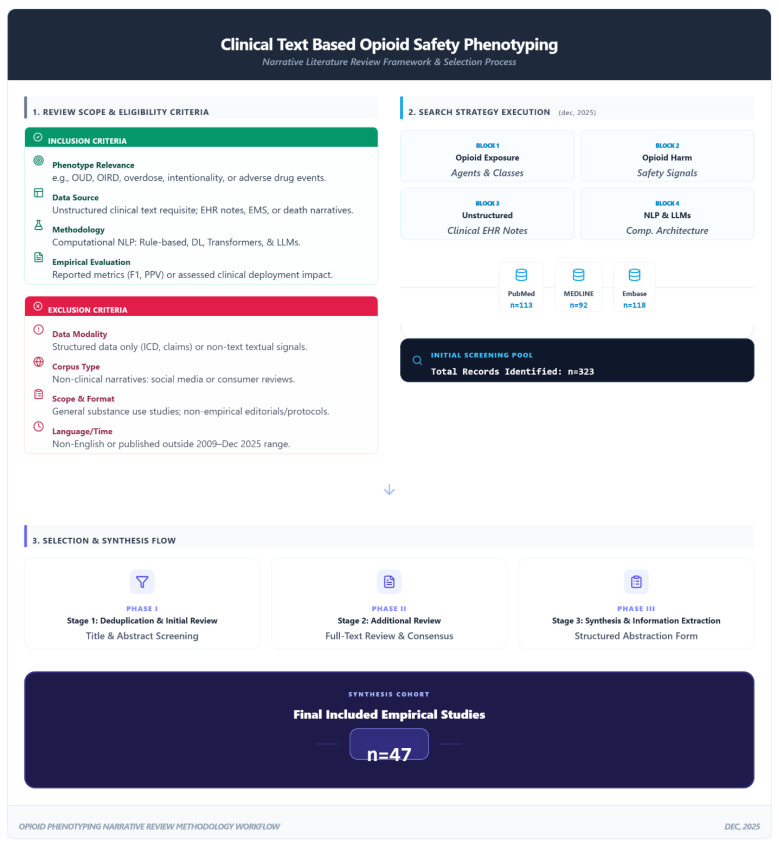
Methodological Framework and Study Selection Workflow for Clinical Text-Based Opioid Safety Phenotyping. Detailed Version: This infographic illustrates the three-tiered interpretive narrative review process. (1) Review scope & eligibility criteria: details the inclusion requirements, specifically focusing on opioid-related phenotypes (e.g., OUD, OIRD), the requisite use of unstructured clinical text, and computational methodologies ranging from rule-based NLP to modern LLM architectures. (2) Search strategy execution: outlines the four-block concept strategy executed in December 2025 across PubMed, Ovid MEDLINE, and Embase, which identified an initial pool of 323 records. (3) Selection & synthesis flow: depicts the systematic filtering process, including independent dual-reviewer screening and full-text consensus resolution, leading to the final synthesis cohort of 47 empirical studies.

This narrative review synthesizes empirical research on opioid-safety phenotyping from clinical texts, focusing on the development and evaluation of computational approaches for identifying opioid-related harms. We define opioid-safety phenotyping as the computational process of identifying both chronic clinical states, such as Opioid Use Disorder, and acute iatrogenic events, such as respiratory depression, within the clinical narrative. In contrast to systematic reviews, which employ stringent inclusion criteria and aim to encompass all relevant literature, our narrative review adopts a more flexible, interpretive approach to capture the evolution of diverse methodologies. We note that, although we used structured eligibility criteria and dual-reviewer screening to improve transparency, our objective was not to conduct a formal scoping or systematic review. Specifically, we did not aim to exhaustively map all study types or outcomes, nor did we perform formal risk-of-bias appraisal across included studies. Instead, we used a narrative approach to support thematic synthesis focused on methodological strata, validation practices, and deployment considerations in safety-critical opioid surveillance [34,35,36,37]. To strengthen transparency while preserving a narrative synthesis approach, we applied structured eligibility criteria and a dual-reviewer screening workflow. By adopting this approach, we prioritized studies that utilized clinical texts as input to identify opioid-related unsafe use patterns and iatrogenic safety signals. The methodological framework and study selection workflow are summarized in Figure 2.

**Inclusion Criteria: Studies were included if they met the following four criteria.**

(a) Phenotype Relevance: The study addressed specific opioid-related harms or safety signals, including opioid use disorder and dependence, problematic prescription opioid use (e.g., aberrant behaviors), overdose or poisoning (including intentionality), opioid-induced respiratory depression, or opioid-related adverse drug events. We also included studies using proxies for harm, such as naloxone administration or addiction medicine consultation triggers, as well as studies that used a clinical-text-derived opioid phenotype to analyze downstream clinical outcomes.
(b) Data Source: The study utilized unstructured clinical texts (used either alone or in combination with structured EHR data) as a primary input. Eligible text sources included EHR clinical notes, such as progress notes, discharge summaries, and Emergency Department (ED) provider documentation, as well as adjacent clinical narratives used in real-world surveillance, including EMS patient care reports and medicolegal death investigation narratives.
(c) Methodology: The study applied computational natural language processing methods, ranging from rule-based and heuristic approaches (e.g., regular expressions and ontology mapping) to traditional machine learning, deep learning, and modern transformer or large language model architectures.
(d) Empirical Evaluation: The publication reported empirical performance metrics, such as sensitivity, positive predictive value, and F1-score, derived from a validation process (retrospective, temporal, or external), or assessed usability and deployment impact in a clinical workflow.

**Exclusion Criteria: Studies were excluded based on the following characteristics.**

(a) Data Modality: Research relying solely on structured administrative data, such as ICD codes, pharmacy claims, or registries, without a text-processing component, or studies where the primary signal was non-textual (e.g., imaging or waveforms).
(b) Corpus Type: Studies analyzing non-clinical corpora, such as social media, consumer reviews, or spontaneous pharmacovigilance narratives unconnected to clinical workflow documentation.
(c) Scope and Format: General substance-use studies lacking specific reporting on opioid-related outcomes, and non-empirical publications such as editorials, perspectives, and protocols without results.
(d) Language and Timeframe: Articles published in non-English languages or published outside the review period of 2009 through December 2025.

### 2.2. Article Selection, Literature Search, and Review

To identify the relevant evidence base, we performed a structured search of PubMed, Ovid MEDLINE, and Embase, with the final query executed in December 2025. The search strategy employed Boolean logic to combine four distinct concept blocks, ensuring broad coverage of the intersection between clinical text and opioid safety:

(a) Opioid Exposure: Terms identifying specific agents (e.g., morphine, fentanyl, oxycodone, methadone, buprenorphine) and general opioid classes.
(b) Opioid Harm and Phenotypes: Keywords targeting specific safety signals, OIRD, overdose or poisoning, naloxone administration, adverse events (e.g., sedation, delirium), and OUD or misuse behaviors.
(c) Data Source: Terms restricting results to unstructured healthcare data, such as EHR, clinical notes, nursing documentation, discharge summaries, and free text.
(d) Methodology: A comprehensive block capturing the evolution of computational methods, ranging from NLP and text mining to machine learning, deep learning, and modern transformer or LLM architectures, including terms like BERT, ChatGPT, and Retrieval-Augmented Generation (RAG).

A typical Title/Abstract Boolean structure for our opioid-safety review was: (opioid* OR morphine OR fentanyl OR oxycodone OR hydrocodone OR related opioid terms) AND (overdose OR poisoning OR OIRD OR naloxone OR OUD OR misuse OR delirium OR related safety terms) AND (“electronic health record*” OR EHR OR “clinical note*” OR “nursing note*” OR “discharge summar*” OR “progress note*” OR related clinical text sources) AND (“natural language processing” OR “machine learning” OR “deep learning” OR transformer* OR “large language model*” OR ClinicalBERT OR ChatGPT OR “retrieval-augmented generation” OR related AI methods).

**Figure 3:**
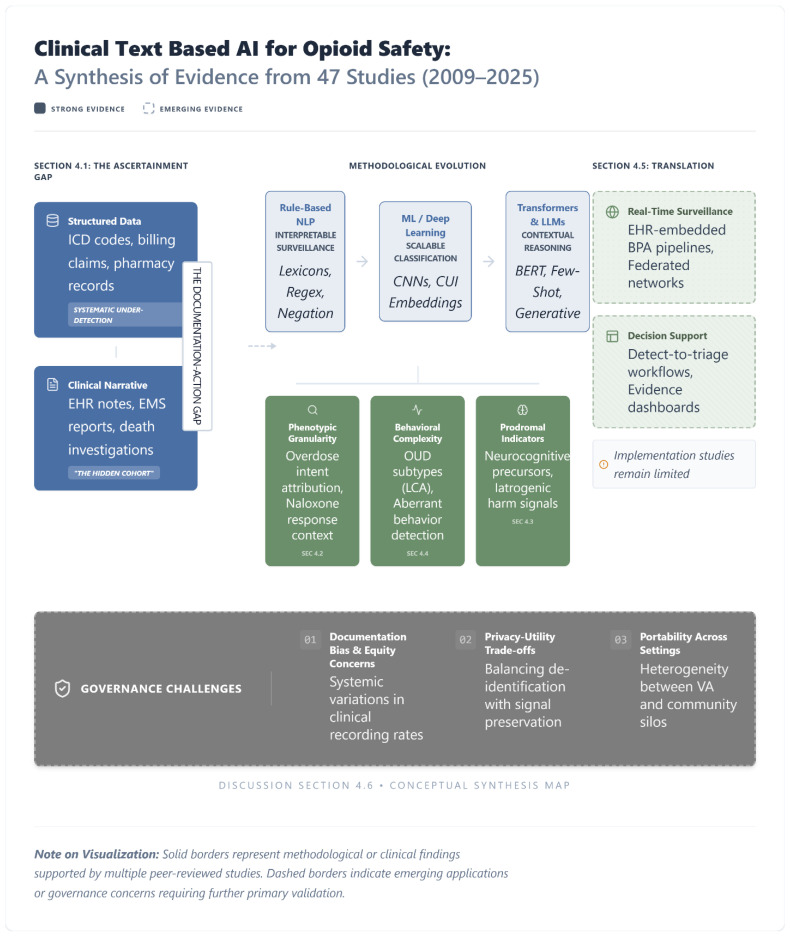
Text-Based AI for Opioid Safety: A Synthesis of Evidence from 47 Studies (2009–2025). This diagram synthesizes the evidence base for natural language processing applied to opioid-related harm detection. The literature addresses a documentation-to-action gap: structured administrative data may under-detect opioid-related harms, leaving a cohort of at-risk patients documented primarily in text. Over 16 years, methods have evolved from rule-based NLP (*n* = 19) to ML/DL (*n* = 18) to transformer architectures (*n* = 10), enabling recovery of granular signals including overdose intent, behavioral complexity, and prodromal indicators of acute harm. Implementation evidence remains limited and uneven, with relatively few studies demonstrating real-time deployment or clinical decision support integration and even fewer evaluating downstream patient-level impact. Governance challenges (including documentation bias, equity concerns, and cross-site portability) have been identified but not yet systematically addressed. Recommendations for bridging these gaps are presented in Section 5 (Future Directions).

Across the three databases, the initial search yielded a total of 323 records (PubMed: 113; Ovid MEDLINE: 92; Embase: 118), including full-text articles, extended abstracts, and pre-prints. Following cross-database deduplication, the study selection process proceeded in two stages: two independent reviewers first screened titles and abstracts for eligibility, followed by a full-text review of potentially relevant records. Disagreements regarding inclusion were resolved through discussion and consensus. This selection process resulted in a final set of 47 empirical studies synthesized in this review. The resulting evidence landscape and methodological evolution across the 47 included studies are summarized in Figure 3.

This review spans a broad set of computational approaches used to detect and characterize opioid-related harms from clinical text. We summarize approaches in three families: rule-based NLP, machine learning and deep learning models trained on labeled data, and transformer or large language model-based systems. We describe how studies typically define phenotypes, construct reference standards, and evaluate performance, because these design choices strongly influence interpretability and real-world usefulness. Throughout, we emphasize practical issues that matter in safety-critical deployment, including calibration, portability across settings, and governance considerations.

## 3. Results: Overview of Selected Articles

The Results are organized to help readers compare what different methodological families can and cannot do for opioid safety surveillance. We first synthesize rule-based systems that prioritize transparency and clinician interpretability, then ML and DL approaches that improve scalability but depend on labeling and validation quality, and finally transformer- and LLM-based approaches that can capture richer context but require careful safeguards. Within each family, we summarize common use cases, typical validation strategies, and where evidence supports workflow impact rather than retrospective accuracy alone. This structure is intended to support both clinical readers looking for operational implications and informatics readers assessing technical rigor.

The final evidence set consists of 47 empirical studies published between 2009 and 2025. These studies were synthesized across three methodological strata aligned with the field’s technical evolution (Table 2, Table 3 and Table 4):

- Rule-based NLP (*n* = 19);
- Traditional ML/DL (*n* = 18);
- Transformer/LLM-based approaches (*n* = 10).

### 3.1. Study Characteristics and Clinical Targets

The majority of these studies were retrospective and conducted within U.S. healthcare environments, with performance typically measured against manual chart review, expert annotation, or structured-code baselines. Across the literature, a few recurring safety targets dominated, including opioid use disorder and dependence; misuse or problematic opioid use, including aberrant behaviors; and overdose or poisoning. Smaller subsets of studies evaluated naloxone administration as a proxy for overdose response, opioid-induced respiratory depression, and neurocognitive symptoms as indicators of opioid-related adverse effects. Across targets, operational definitions and clinical contexts varied substantially, contributing to heterogeneity in reported performance and comparability across studies.

### 3.2. Data Sources

While EHR narratives (such as ED/inpatient notes and discharge summaries) were the dominant text inputs, the review also identified use of real-world surveillance data. This included EMS patient-care narratives, medicolegal death investigation narratives, and public datasets like MIMIC. Several studies also paired text with structured EHR elements (e.g., medications, diagnoses, or vitals) to support multimodal phenotyping and prediction.

### 3.3. Methodological Findings by Stratum

In this section, we summarize methodological findings across three broad strata of approaches used for opioid safety surveillance from clinical text. We first describe rule-based NLP systems, which are typically transparent and straightforward to govern but can require ongoing maintenance to address documentation drift and site-specific language. We then synthesize machine learning and deep learning models, which can improve scalability and performance in some settings but are highly dependent on phenotype definitions, labeling quality, and external validation. Finally, we review transformer- and large language model-based approaches, which can capture richer clinical context and support more structured extraction, but require careful safeguards to ensure outputs remain evidence-grounded, calibrated, and appropriate for safety-critical workflows.

Across included studies, reference standards for opioid-related phenotypes varied substantially, including manual chart review or clinician adjudication, administrative code-based definitions, instrument-anchored labels (e.g., Addiction Behavior Checklist, Opioid Risk Tool), and proxy outcomes such as naloxone administration or consult triggers. Because these strategies capture different clinical constructs and operate at different levels of certainty, cross-study comparisons of performance should be interpreted cautiously. In particular, proxy and code-based labels may introduce misclassification, and differences in case mix and prevalence across settings can contribute to spectrum effects that influence apparent discrimination and predictive value.

Rule-based NLP (Table 2): These studies emphasized interpretable surveillance and operational phenotyping. Common techniques included lexicons, regular expressions, and negation filters. A recurring finding was that text-based rules could recover clinically documented cases that were entirely missed by traditional ICD-only workflows. This stratum most often prioritized transparency and feasibility, but portability and maintenance under documentation drift were recurring practical considerations.

ML/DL (Table 3): This group was dominated by supervised classification for OUD and overdose phenotypes, often integrating structured EHR variables with text features mapped to UMLS concepts. Notable advancements in this group included weak supervision to reduce labeling burdens and audits for model bias and portability. A subset of studies additionally reported workflow-facing endpoints (e.g., alert-triggered actions, consult uptake, or efficiency metrics), reflecting early steps toward implementation.

Transformer/LLM-based (Table 4): Representing the most recent wave of research, these studies utilized transformer classifiers and generative decoder-only LLMs for information extraction and classification in complex clinical corpora. This stratum also highlighted efforts to build expert-annotated benchmarks to standardize model comparisons. Relative to prior epochs, this stratum more frequently emphasized standardized evaluation datasets and richer extraction schemas, though external validation and calibration remained inconsistently reported.

### 3.4. Evaluation Practices and Reference Standards

Across the 47 studies, evaluation most commonly relied on retrospective, single-site designs using manual chart review or expert adjudication on sampled notes or encounters, often with ICD codes as a baseline comparator rather than a gold standard. Reference standards ranged from clinician review to instrument-anchored definitions (e.g., addiction behavior checklist (ABC)- or opioid risk tool (ORT)-derived labels) and proxy triggers (e.g., naloxone administration, consult triggers, or mortality linkage), depending on the safety target. Validation was most often internal (cross-validation or held-out testing), while temporal splits, external validation across institutions, calibration reporting, and workload-oriented endpoints (e.g., number needed to evaluate or chart review time) were less frequently reported. Across studies, reference standards and validation strategies were highly variable, external validation was uncommon, and calibration, threshold selection, and error analyses were rarely reported, which constrains confidence in real-world transportability and thresholded clinical use.

## 4. Discussion

The 47 studies synthesized in this review suggest that clinical notes are not merely hidden data but a clinically actionable source of opioid safety signals that structured administrative records often overlook. Over 16 years of methodological evolution, most studies remain retrospective, with a small subset offering early examples of real-time clinical decision support. At the same time, substantial gaps persist between model discrimination performance and demonstrated patient-level impact. Methodologically, the evidence base is dominated by retrospective studies conducted within U.S. healthcare environments, with performance typically evaluated against manual chart review, expert annotation, or structured-code baselines. A smaller subset reports external validation and workflow-relevant utility metrics, such as positive predictive value and number needed to evaluate, which more directly reflect clinical workload and actionability.

### 4.1. Addressing the Under-Ascertainment of OUD and Misuse

A recurring theme across the literature is the discrepancy between patients with clinically documented opioid-related concerns and those with a formal administrative diagnosis. This ascertainment gap suggests that relying solely on structured ICD-10 codes can lead to underestimation of opioid-related harms within health systems. Text-based AI has been repeatedly used to surface this documentation-defined cohort, with early rule-based logic showing that NLP dictionaries could recover patients with problematic use behaviors, thus supporting automated surveillance [39,55,72]. Subsequent machine learning and deep learning approaches expanded this scalability [57,62,82,83], demonstrating that neural models could ingest large volumes of notes to flag OUD risk with high precision in specific settings. However, the clinical meaning of documentation-derived OUD and misuse phenotypes depends on operational definitions, context, and adjudication standards, and comparable performance across settings is not consistently demonstrated. In addition, documentation-derived cohorts [56] may exhibit different comorbidity profiles than those captured by ICD codes, implying that case-finding choices can influence epidemiologic estimates and downstream analyses. In emergency department populations, some studies report supervised classifiers that exceed ICD-only baselines for misuse detection, including high precision in one evaluation [82]; however, reported performance varies by phenotype definition, documentation context, and validation design.

### 4.2. Enhancing Phenotypic Granularity: Overdose Intent and Context

A second theme is the use of clinical text to recover context for acute adverse events, details that are often lost in administrative data. Identifying an overdose is a coarse signal, and safety surveillance frequently requires distinguishing intent, circumstance, and the substance involved. Rule-based systems laid the groundwork for more granular extraction [45,46], showing that NLP could distinguish suicidal intent from accidental poisoning in curated settings. In the pre-hospital setting, text mining of EMS narratives can identify naloxone administrations missed by structured fields [44], recovering details on dosing and patient response. Similar context recovery has been reported in pediatric populations [48], mortality narratives [76], and hospital-wide databases [52]. As methods advanced, machine learning models began to address variability across documentation styles [63,71], and more recent work has explored transformer- and LLM-based classification in complex narrative corpora [75,76]. Across these studies, a consistent value of text is not only case detection but also contextual enrichment that can support review and routing. Nevertheless, the degree to which context extraction changes clinical decisions or outcomes is infrequently evaluated directly.

### 4.3. The Prognostic Frontier: Prodromal Signs and Iatrogenic Harm

A distinct subset of studies focused on identifying acute iatrogenic harms, including opioid-induced respiratory depression, where narrative documentation may precede structured failure signals and offer a potential window for intervention. Recent studies report associations between neurocognitive symptoms documented in clinical notes and subsequent overdose events in specific cohorts [47,50]. Methodologically, this line of work increasingly uses sequence modeling and weak supervision, in which expert-defined labeling functions search for narrative clues, such as naloxone effectiveness, to train models for OIRD detection [64]. Prognostic applications have also incorporated clinical text into multimodal risk models [68,83]. Similarly, LLM-assisted feature selection has been explored to refine text-derived predictors in inpatient settings [78]. However, these studies also highlight that OIRD and oversedation are low-prevalence, high-stakes phenotypes where errors have asymmetric consequences, and where reference standards and operating thresholds strongly shape apparent performance. Many studies evaluate retrospective prediction rather than prospective intervention, limiting causal inferences about clinical benefit.

### 4.4. Characterizing Heterogeneity Through Subtyping and Integration

Several studies emphasize that opioid misuse is heterogeneous, motivating unsupervised and semi-supervised approaches to identify distinct subtypes. Latent class analysis [58] and clustering approaches [61] have been used to describe misuse profiles with differing utilization patterns. To represent risk more continuously, text-derived classifiers/flags have been developed [41,65], and specialized annotation schemas have been used to extract social and behavioral determinants of health [66,80]. These approaches may better preserve clinical nuance than binary labeling, but subtype definitions and clinical actionability vary across studies and settings, limiting direct comparability. Documentation context also remains central: the same textual cue may reflect historical history, active concern, or diagnostic uncertainty, which can increase mislabeling risk without clinician adjudication.

### 4.5. Real-World Implementation: Workflow and Usability

A smaller, implementation-oriented subset of studies has shifted focus from offline accuracy to workflow integration and clinician workload. In these studies, systems are most commonly positioned as detect-to-triage tools, flagging high-risk cases for human adjudication. However, this reflects an adoption pattern more than a settled best practice, because most evaluations remain retrospective and only a smaller subset reports prospective or workflow impact endpoints. Examples include EHR-integrated Best Practice Alert pipelines [57,75] embedded in Epic workflows and applied to inpatient and emergency department notes, with external validation and operational metrics reported in deployed settings. For example, Afshar et al. [75] report external validation with PPV and number needed to evaluate and note that LLM testing did not add benefit in that deployment context. The multi-site Opioid Overdose Network (O2-Net) [84] illustrates a federated infrastructure paired with an EHR documentation tool, reporting template uptake and increased naloxone prescribing. Usability-oriented work also emphasizes returning reviewable evidence from text, such as pharmacovigilance prototypes [73] and large-scale entity extraction pipelines [81]. Across implementations, a clinically credible pattern is the combination of a case flag with transparent supporting evidence and explicit thresholds that reflect acceptable workload and harm trade-offs. Although detect-to-triage is the dominant operational posture in implementation-oriented studies, longitudinal safety surveillance represents a distinct use case, in which text-derived signals are tracked over time to characterize evolving risk and recurrent events across encounters. The evidence base for longitudinal surveillance remains more limited, and comparative evaluations of sustained monitoring strategies, alert frequency, and escalation pathways are uncommon. Even in deployed examples, generalizability beyond the implementation environment and prospective outcome evaluation remain limited.

### 4.6. Ethical Stewardship: Equity, Privacy, and Portability

The high stakes of opioid therapy, including risks of stigmatization and undertreatment of pain, make ethical governance a central theme of this review. Fairness audits [69] have documented subgroup performance disparities, reinforcing the need for mitigation, recalibration, and routine subgroup monitoring. The challenge of capturing risk behaviors across fragmented documentation silos, such as between VA and community settings [70,74], has also motivated hybrid approaches combining rules with supervised classifiers. To facilitate safer sharing, PHI-reduced concept-based representations [67] have been shown to preserve predictive performance while easing governance barriers. Documentation drift and cross-setting portability further motivate external validation and calibration, which remain inconsistently reported across the literature. Given these risks, the strongest deployment rationale supported by the current evidence is to preserve clinician adjudication and avoid automated labeling that could restrict appropriate care.

### 4.7. Limitations

Several limitations of this review should be acknowledged. First, the evidence base is dominated by retrospective studies conducted largely within U.S. healthcare systems, which may limit generalizability to international settings, non-English documentation environments, and prospective deployment contexts. Second, substantial heterogeneity in phenotype definitions precludes formal meta-analysis and complicates cross-study comparison. Terms such as “misuse,” “aberrant behavior,” and “problematic use” were inconsistently operationalized, and studies rarely distinguished between patients with cancer-related pain and those with non-cancer chronic pain. These populations differ substantially in opioid prescribing norms, risk–benefit considerations, clinical trajectories, and the potential harms of surveillance-driven labeling. In oncology, opioid therapy is often clinically appropriate, and false-positive misuse flags may carry distinct downstream consequences (e.g., stigma in the record, barriers to analgesia, or inappropriate care escalation) compared with non-cancer settings where prescribing is often more tightly governed by stewardship and monitoring policies. Stratification by cancer status was uncommon, and many studies drew from mixed or unspecified populations. This limits our ability to determine whether clinical-text-based AI models perform differently or have different clinical implications across contexts, and whether surveillance tools require population-specific thresholds or deployment strategies. Third, reporting and validation practices were uneven. Reference standards varied widely, ranging from manual adjudication to proxy labels, and external validation was uncommon. Few studies provided deployment-relevant operating characteristics such as calibration metrics, threshold selection rationale, or error analyses that quantify the likely false-positive burden in practice. Fourth, while equity is a critical concern in opioid surveillance, few studies reported subgroup performance by race, ethnicity, or socioeconomic status, limiting our ability to assess algorithmic fairness across the field. Finally, a major interpretability constraint is that “ground truth” varies by study. Performance metrics are therefore not directly comparable across studies using different reference standards, and reported estimates may reflect misclassification and spectrum effects as much as algorithmic capability. These limitations collectively define the evidence gaps that must be addressed before text-based AI can be safely adopted in clinical workflows. Section 5 presents a roadmap of practical priorities to address them.

## 5. Future Directions: A Roadmap for Safe Adoption

**Figure 4:**
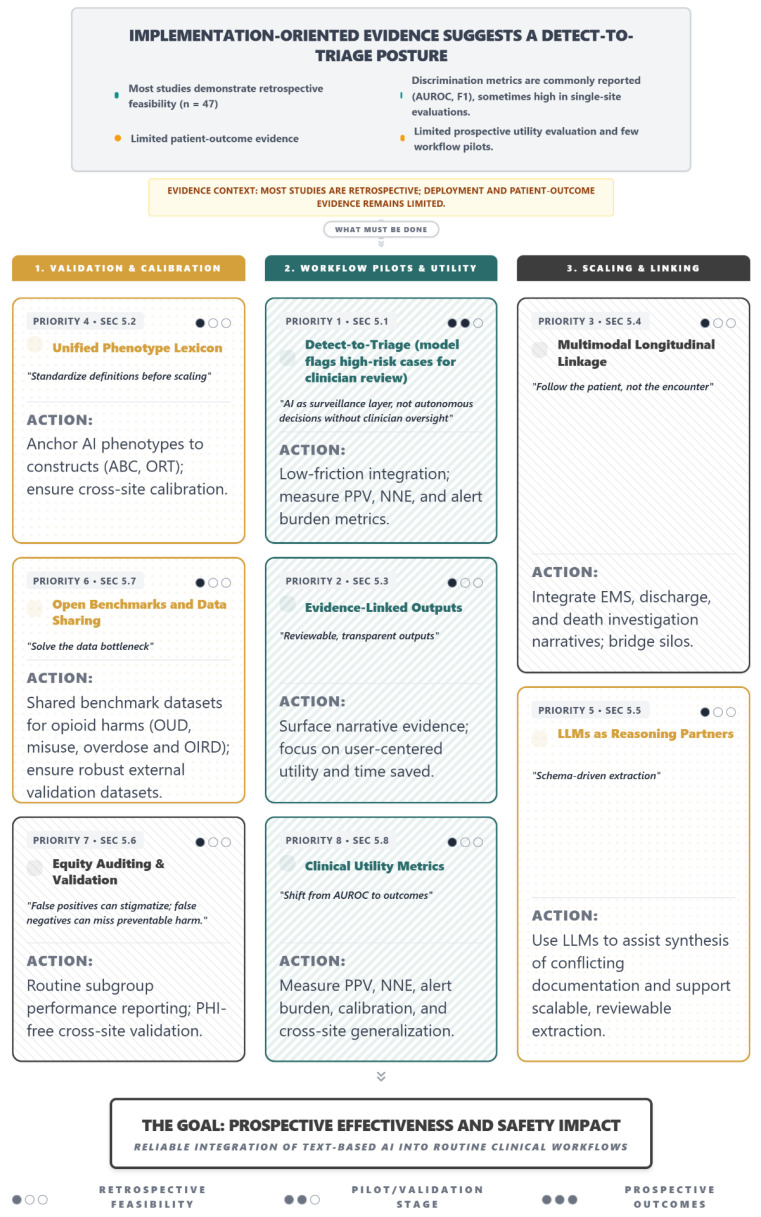
A Roadmap for Safe Adoption: Eight Priorities for Text-Based Opioid Safety AI. Although 47 studies demonstrate retrospective feasibility for detecting opioid-related harms from clinical text, substantial gaps remain before these tools can be reliably integrated into routine clinical workflows. This roadmap summarizes eight priorities aligned with three implementation domains. Validation and Calibration (Priorities 4, 6, and 7) emphasizes standardized phenotype definitions, shared benchmarks and data sharing, and equity auditing with cross-site validation. Workflow Pilots and Utility (Priorities 1, 2, and 8) focuses on detect-to-triage workflows, evidence-linked outputs that surface reviewable narrative support, and evaluation using clinical utility metrics such as PPV, number needed to evaluate, and alert burden. Scaling and Linking (Priorities 3 and 5) highlights multimodal longitudinal linkage across care settings and the role of large language models as reasoning partners to support scalable, reviewable extraction. Together, these priorities outline a pathway from retrospective feasibility toward prospective validation and measurable safety impact. This figure is organized by implementation domain rather than strict chronology. The priority numbers indicate a recommended sequence across domains (Priority 1 through Priority 8), and readers may follow the priorities in numerical order while using the domain headings to interpret the intended focus of each item.

Despite these advances, the evidence base reveals persistent gaps that should be addressed to support reliable integration of text-based opioid safety tools into clinical workflows. Specifically, the literature is dominated by retrospective studies conducted largely within U.S. systems, phenotype definitions remain inconsistent across studies, and subgroup performance reporting is uncommon despite equity being central to opioid surveillance. We therefore turn now to a roadmap for safe adoption that addresses these challenges (Figure 4).

### 5.1. Shifting the Paradigm: From Passive Surveillance to Active Clinical Stewardship

The current body of evidence suggests that detect-to-triage is a conservative and currently best-supported implementation posture, not a definitively proven endpoint. Prospective studies should explicitly compare alternative workflow designs and thresholds, and evaluate patient-centered and process outcomes to determine when, where, and for whom detect-to-triage provides net benefit. For example, hospital-wide screening systems, such as those described by Afshar et al. [57] and Afshar et al. [75], demonstrate that success depends not only on model choice but also on workflow design. Specifically, effectiveness is influenced by whether AI flags are review-ready and directed to clinical teams with a clear pathway for intervention, such as addiction medicine or toxicology consultation. Future implementations should prioritize low-friction integration where the AI output is treated as a prompt for human review, effectively centering clinical expertise on the most complex cases identified from the narrative.

For clinical deployment, we suggest a minimum evaluation framework that complements AUROC and F1 with measures that directly map to workflow and safety. At a minimum, studies should report (1) an explicit reference standard and phenotype definition, (2) discrimination and class-imbalance robust metrics (e.g., AUPRC) alongside calibration (calibration plot and calibration slope/intercept or Brier score), (3) a prespecified operating threshold with rationale, including decision-curve style utility or a cost-sensitive analysis when feasible, (4) workload and actionability metrics such as positive predictive value and number needed to evaluate at the chosen threshold, (5) external validation across sites or time with monitoring for documentation drift, and (6) prospective or quasi-prospective evaluation of workflow endpoints (e.g., time to review, consult placement, naloxone prescribing, or avoided adverse events) whenever the model is intended to trigger clinical action.

Acceptable operating points vary by setting because prevalence, downstream actions, and tolerance for false positives differ. In the emergency department, where overdose and intent assessments are time-sensitive, higher sensitivity with transparent evidence spans may be acceptable if false positives are routed to rapid human review, and workload should be reported explicitly using PPV and number needed to evaluate. In inpatient surveillance for oversedation or opioid-induced respiratory depression, false negatives carry substantial harm, so thresholds may prioritize sensitivity and continuous monitoring, but calibration and alert burden are critical to prevent alarm fatigue. In pain clinics and chronic opioid therapy populations, where prevalence of acute events is lower and decisions may affect long-term access to analgesia, specificity, fairness auditing, and clear separation of historical versus active risk are essential, and models should be evaluated for longitudinal stability and portability across documentation styles.

Operational challenges commonly include alert fatigue, unclear ownership of alerts, and variability in clinical follow-through after an alert is generated. Practical solutions include non-disruptive integration into the EHR, explicit routing to accountable teams (e.g., addiction medicine, toxicology), and presenting reviewable evidence from text to support rapid adjudication and appropriate action. Where available, workload tracking (e.g., number needed to evaluate) should be monitored over time to ensure sustainability.

Recent guidance for clinical AI evaluation emphasizes that real-world decision support requires reporting beyond discrimination metrics, including workflow integration, operating thresholds, calibration, and monitoring in live settings [27,85,86,87,88].

### 5.2. The Definitional Crisis: Toward a Unified Lexicon of Opioid Harm

One of the most significant barriers to the scalability of these tools is the heterogeneity of phenotype definitions across the literature. Terms such as “misuse,” “aberrant behavior,” and “problematic use” are frequently operationalized in inconsistent ways, ranging from ICD-code proxies to granular behavioral checklists. Progress is likely to remain constrained without a unified clinical lexicon. Future research should prioritize anchoring AI phenotypes to established clinical constructs, such as the addiction-behavior checklist or opioid-risk tool, as seen in interpretable, rule-based automation efforts of Chatham et al. [40] and Haller et al. [43]. By aligning model outputs with the language clinicians already use, we can improve both the transportability of models and the reliability of multi-site surveillance.

### 5.3. Design for Trust: Evidence-First and Temporal-Aware AI

For AI to be trusted at the bedside, its outputs must be reviewable and transparent. We recommend that future systems be designed with an “evidence-first” architecture; rather than providing a single opaque risk score, models should present the specific “evidence spans” within the clinical narrative that triggered the alert, along with explicit operating thresholds and calibration assessment when feasible. This is particularly critical in safety-critical settings like the ICU or emergency department, where the response to naloxone or suspected intent documented in text can change the entire course of care. Furthermore, models must become “temporal-aware,” distinguishing between a patient’s historical OUD diagnosis and an active, acute safety event like opioid-induced respiratory depression. As Sung et al. [50] and León et al. [47] have reported, capturing the temporal ordering of symptoms is essential for identifying prodromal warning signs that may precede severe outcomes.

### 5.4. Breaking the Silos: Multimodal Continuity Across the Patient Journey

Opioid-related harms do not occur in isolation; they are trajectories that span fragmented care settings. Future work should treat linkage across time and setting, from EMS narratives to discharge summaries and death investigations, as a primary design goal. The work of Harris et al. [44] and Funnell et al. [76] highlights the value of narrative data from outside the traditional EHR for building a more complete picture of patient risk. In this framing, longitudinal surveillance complements detect-to-triage by supporting ongoing monitoring of risk trajectories rather than single-episode case identification, but robust evidence on optimal monitoring intervals, escalation criteria, and downstream outcomes remains sparse. Future evaluations should examine hybrid models that combine structured physiological data with multi-source narrative meaning to support a longitudinal safety record that follows the patient across the continuum of care. This continuity is essential for identifying risks faced by vulnerable populations, such as veterans receiving care across both VA and community silos, while also testing portability across institutions and documentation practices. Comparative evaluations across settings and documentation styles remain limited, and portability should be treated as an empirical question requiring external validation and recalibration.

### 5.5. The Generative Frontier: LLMs as Reasoning Partners, Not Just Classifiers

The advent of LLMs offers the potential to extend classification toward “schema-driven extraction.” While earlier rule-based and ML/DL systems were often brittle to documentation changes, modern transformer architectures, such as those evaluated by Kwon et al. [79] and Harel-Canada et al. [77], can capture clinical nuance relevant to rare or context-dependent signals. However, the reliability and drift of these systems must be managed explicitly, and outputs should remain reviewable and attributable to underlying text evidence. Future research should explore using LLMs not just to flag a case, but as clinician-facing assistants that can synthesize documentation into structured summaries for adjudication, as seen in the work of Paredes et al. [81] and Sorbello et al. [73], while explicitly evaluating error modes, calibration, and cross-site transportability.

Although transformer- and LLM-based approaches are increasingly studied, evidence of consistent real-world superiority over encoder-based or rule-based methods remains limited, and comparative gains appear task-dependent. LLMs may add the most value when the goal is schema-driven extraction and contextual synthesis that produces clinician-reviewable outputs for adjudication, particularly in heterogeneous narratives where rigid lexicons can be brittle and where evidence spans and structured summaries facilitate review [73,81]. In contrast, for well-specified detection or classification tasks with stable documentation patterns and clearly defined labels, encoder-based models and rule-based approaches can remain competitive, often with simpler governance and more predictable failure modes; in deployed workflows, some evaluations have reported limited incremental benefit from adding LLM testing beyond existing pipelines in that implementation context [75]. Accordingly, current evidence most strongly supports LLMs as clinician-facing assistants that support review and routing, rather than as autonomous decision-makers in safety-critical opioid surveillance.

A related limitation of transformer-based and large language model-based systems is the potential for hallucinations, defined here as outputs that appear plausible but are not supported by the underlying clinical text or the external evidence base. In opioid safety surveillance, this risk is most relevant when models are used for open-ended generation, summarization, or natural language justification, and it can also occur in extraction settings if prompts permit unconstrained inference beyond the documented record. Practical mitigations include schema-constrained output formats, span-grounded extraction with explicit provenance, retrieval-augmented generation to tether responses to retrieved evidence, rule-based post-processing checks, and clinician review for high-consequence decisions, particularly when outputs may trigger safety actions or escalation pathways [89,90,91,92].

We therefore distinguish research utility (rapid prototyping, flexible extraction, and hypothesis generation) from clinical readiness, which requires evidence-grounded outputs, calibration and threshold justification, drift monitoring, and prospective workflow evaluation.

### 5.6. Mandatory Accountability: Equity Auditing and External Validation

The asymmetric consequences of error in opioid surveillance, where false positives can stigmatize and false negatives can delay harm-reduction opportunities, demand a higher standard of accountability. External validation and equity auditing should be considered key prerequisites before any text-based model is deployed in a clinical workflow. Equity considerations in opioid safety surveillance extend beyond average performance metrics because the consequences of false positives and false negatives are not symmetric and may differ across subgroups. False positives can amplify stigma, trigger disproportionate monitoring, or contribute to unintended restriction of analgesia, particularly in populations historically subject to biased documentation and undertreatment of pain, whereas false negatives can delay naloxone provision, consultation, or escalation in high-risk patients. Within detect-to-triage frameworks, these risks can be mitigated by requiring that alerts present reviewable evidence spans and clear rationale, by explicitly separating historical from active safety signals, and by using standardized adjudication protocols that prohibit automated labeling or medication restriction based solely on model output. Minimum governance practices should include routine subgroup reporting at the deployed operating threshold (not only overall AUROC), monitoring differential alert burden and downstream actions across groups, periodic recalibration and drift checks, and oversight mechanisms to review harms and update thresholds, lexicons, and workflows when disparities emerge. As Thompson et al. [69] demonstrated, documentation bias can translate into algorithmic bias across racial and ethnic subgroups. Future studies should routinely report calibration metrics and subgroup performance, ensuring that models are not only accurate but also fair at operating thresholds that reflect clinical workload and risk tolerance. Institutions must also address the privacy–utility trade-off by adopting protected-health-information-free representations that allow for safe, cross-site model validation and sharing. In practice, privacy-preserving strategies may include PHI minimization (e.g., concept-based or de-identified representations), role-based access controls and auditing, and restricting raw-text access to approved workflows under institutional governance and data-use agreements. When feasible, institutions should also audit whether model-triggered actions (e.g., consult placement, naloxone ordering, pain regimen changes) differ across subgroups after controlling for clinical context, to detect unintended restrictions of appropriate care.

### 5.7. Solving the Data Bottleneck: Open Benchmarks and Privacy-Preserving AI

Progress in this field has been hampered by the inability to share sensitive clinical notes. To accelerate reproducible research, we should invest in the creation of open-source benchmarks, such as those derived from de-identified critical-care databases like MIMIC. Recent efforts by Kwon et al. [79] and Harel-Canada et al. [77] to release expert-annotated datasets for opioid-related signals are essential first steps. We recommend the development of a shared “benchmark repository” that spans multiple safety phenotypes, including OUD, overdose, and OIRD, to allow researchers to compete on standardized, clinically meaningful tasks, while also supporting federated or privacy-preserving evaluation when note sharing is not feasible. Future evaluations should measure patient-centered outcomes, such as overdose-related ED revisits, naloxone prescribing and uptake, and opioid-related adverse events, in addition to process measures like time to review and consult placement.

### 5.8. Measuring What Matters: Shifting from AUROC to Clinical Utility

Finally, we must redefine what “success” looks like for clinical AI. High AUROC scores in a retrospective lab setting do not guarantee safety at the bedside. Evaluations should move toward endpoints that reflect real-world clinical utility, such as chart-review time saved, reduction in alert fatigue, and number needed to evaluate, reported alongside thresholded sensitivity and false-positive burden. More importantly, we need prospective studies that measure downstream impact on patient safety, such as changes in clinical routing, timely linkage to addiction treatment, or reductions in preventable adverse events associated with earlier detection. Broader integration will likely require evidence that these systems improve care processes and patient-centered outcomes under real-world constraints.

## 6. Conclusions

Clinical text-based AI has progressed from early rule-based surveillance toward machine learning- and transformer-based approaches that can identify opioid-related harms and safety signals documented in narrative notes. Across the 47 empirical studies synthesized in this review, text-driven methods frequently identify cases and contextual details that may be missed when surveillance relies solely on structured administrative fields. At the same time, the overall evidence base remains dominated by retrospective, single-site evaluations, with heterogeneous phenotype definitions, inconsistent reporting of calibration and subgroup performance, and relatively limited workflow-integrated and prospective outcome evaluation.

Accordingly, the most defensible near-term clinical role supported by the current, largely retrospective literature is detect-to-triage decision support rather than autonomous diagnosis. This should be viewed as a pragmatic bridge from case-finding to clinical action, pending prospective studies that test optimal workflow integration, calibration at operational thresholds, and downstream impact. In this context, systems flag candidate cases from narrative documentation, surface reviewable supporting evidence, and prioritize clinician adjudication and appropriate routing. This framing aligns model capabilities with the sociotechnical realities of opioid care, where documentation context is variable and errors can carry asymmetric clinical and ethical consequences, including stigmatization and inappropriate restriction of analgesia.

Future progress will depend less on incremental gains in retrospective discrimination metrics and more on establishing shared phenotype definitions, robust external validation, calibration at deployment thresholds, and routine equity auditing. Equally important will be prospective evaluations that quantify workflow impact and patient-centered outcomes, such as reductions in preventable harm through earlier identification and appropriate clinical routing. With evidence-first design and accountable governance, clinical-text-based AI may become a practical component of opioid safety stewardship, narrowing the documentation-to-action gap while preserving clinician oversight.

## Figures and Tables

**Table 1:** The Documentation-to-Action Gap: Clinical Narrative vs. Administrative Data. This table illustrates the under-ascertainment gap in which critical safety signals recorded in text are lost in structured EHR fields.

Clinical Safety Domain	Representative Clinical Narrative Signal	Administrative/Structured Representation (“Data Shadow”)	The Ascertainment Gap
Iatrogenic Oversedation/opioid-induced respiratory depression (OIRD)	“Patient found somnolent with RR 6; briskly rousable following 0.4 mg Narcan.”	Often absent or coded as generic “Respiratory Failure” without drug attribution.	Under-ascertainment of causality: Misses the vital link between opioid administration and the respiratory event.
Opioid Use Disorder (OUD)	“Patient admits to taking more oxycodone than prescribed to cope with stress.”	Frequently lacks a formal OUD International Classification of Diseases (ICD)-10 code during acute encounters.	Under-ascertainment of prevalence: Excludes patients from safety cohorts and prevents longitudinal tracking.
Aberrant Behaviors	“Reports ‘doctor shopping’; urine tox positive for non-prescribed fentanyl.”	Usually unrecorded in structured fields or stored in non-searchable PDFs.	Under-ascertainment of risk: Prevents proactive risk stratification and the deployment of targeted clinician alerts.
Overdose Intentionality	“Notes indicate patient took entire bottle… with suicidal intent.”	Often coded as generic “Opioid Poisoning” without distinguishing intent.	Under-ascertainment of clinical context: Hinders the ability to route patients to appropriate psychiatric or social services.

**Table 2:** Summary of 19 empirical studies utilizing lexicons, regular expressions, and rule-driven logic to identify opioid-related safety signals.

Study	Objective/Phenotype	Setting & Data	Model/Method	Key Performance Metrics	Clinical Advancement
Brown et al., 2024 [38]	Opioid involvement & opioid overdose (validation of Enhanced Opioid Identification Algorithm)	2016 NHCS (U.S. ED/inpatient); stratified validation sample *n* = 900 encounters (unweighted); notes available for NLP *n* = 344 (38.2%)	Rule-based algorithm combining structured medical codes + rule-based NLP on clinical notes; compared to gold-standard medical record abstraction	Opioid involvement: F1 0.95; Overdose F1 0.48	Validates NHCS opioid-identification algorithm performance; highlights data completeness/note availability as a key constraint and motivates refinement for surveillance use
Carrell et al., 2015 [39]	Problem opioid use (clinically documented addiction/abuse/misuse/overuse) among chronic opioid therapy patients	Group Health (Washington State); EHR cohort *N* = 22,142 (2006–2012)	Rule-/dictionary-based NLP (1288-term lexicon; negation/qualification handling) + computer-assisted manual validation of NLP-flagged notes	ICD-9 prevalence 10.1% (2240/22,142); NLP-only additional 728 patients (~3.3%); combined (ICD-9 or NLP) prevalence 13.4%	Semi-automated surveillance from clinical text can raise prevalence estimates by ~one-third vs. codes alone
Chatham et al., 2025 [40]	Problematic opioid use (automated Addiction Behaviors Checklist [ABC] score; compared vs. OUD ICD codes; validated vs. manual review)	VUMC Synthetic Derivative chronic pain cohort (*n* = 8063) + external validation Geisinger interventional pain clinic cohort (*n* = 100)	Rule-based NLP (regular expressions): 27 RegEx operationalizing the 20 ABC items (with opioid-term proximity filtering + negation handling)	Geisinger: F1 0.70, AUC 0.86 (vs manual review) (also reported: VUMC F1 0.73, AUC 0.82; ICD codes lower at both sites)	Interpretable, scalable EHR-note-based alternative that outperforms ICD codes and generalizes across health systems.
Schirle et al., 2021 [41]	Continuum of problematic opioid use/OUD risk (continuous scores)	VUMC BioVU (*n* = 88,869); development subcohorts incl. PheWAS *N* = 29,868 and chronic pain subset *N* = 5697; hold-out test set *n* = 100 (manual review *n* = 99)	Two data-driven scores: (1) PheWAS-derived comorbidity score (weighted phecode phenotypes), (2) NLP text-based score using UMLS CUIs + TF-IDF	AUC vs. manual review: Text-based 0.79; Comorbidity 0.76	Supports EHR-based risk representation on a continuum (beyond binary case/control), enabling scalable screening/decision-support concepts
Haller et al., 2015 [42]	Opioid risk/aberrant drug-related behavior (ADRB) risk screening using ORT, DIRE, and SOAPP	Retrospective EHR cohort of chronic non-cancer pain patients with ≥1 opioid agreement (2007–2012); *N* = 3672	Structured EHR data queries + NLP-based extraction from unstructured text to populate risk-tool items	ORT: moderate/high risk were 2.2×/4.8× more likely to have future ADRBs vs. low risk; DIRE < 14 was 2.9× more likely vs. ≥14	Demonstrates feasibility of automating EHR-based opioid-risk screening to support clinician assessment before initiating opioid therapy
Haller et al., 2017 [43]	Agreement violations & selected ADRBs (early refill, lost/stolen meds, alcohol abuse, illicit drug use); automated ORT risk stratification	Essentia Health (northeastern MN/northwestern WI); retrospective Epic EHR cohort of chronic noncancer pain adults with an opioid agreement (2007–2012); *N* = 3668	Structured EHR database queries + regular-expression NLP on clinical notes (keyword/phrase sets with negation/uncertainty handling); manual validation	Opioid agreement violation detection vs. manual review: Sensitivity 96.1%, Specificity 92.8%, AUC 0.945 (also reports PPV 92.6%, NPV 96.6%)	Automates ORT-based risk stratification and opioid agreement-violation surveillance to support screening/monitoring in chronic opioid therapy
Harris et al., 2025 [44]	Naloxone administration identification from EMS records (structured fields + narratives)	Kentucky statewide EMS database; random sample of 30,000 records (2019)	RegEx detection in narratives + NLP extraction of route/dosage; manual review of structured-text disagreements; combined indicator	115 additional cases (26.3% increase vs. structured alone); RegEx precision 0.94, recall 0.93 (FP = 31, FN = 23); route captured 246 (47.4%) and dosage 358 (69.0%) in narrative-identified cases	Reduces undercounting of naloxone utilization/overdose response in EMS surveillance and adds route/dosage context from narratives
Hazlehurst et al., 2019 [45]	Opioid overdose identification and subtype classification (e.g., intentionality/self-harm; substances)	Kaiser Permanente Northwest (KPNW) development + validation; Kaiser Permanente Washington (KPW) portability test; validation evaluated on *n* = 710 records with machine-readable notes	MediClass NLP (rule-based knowledge module/context rules)	KPNW validation: Any overdose Sens 0.78, Spec 0.89; Intentional overdose Sens 0.74, Spec 0.97	Enables automated EHR-based overdose surveillance and classification of overdose intent/type
Hazlehurst et al., 2016 [46]	Opioid overdose intent (classify intentional overdose/suicide attempt vs. unintentional overdose)	Kaiser Permanente Northwest (KPNW) EHR clinical notes; 493 overdose cases (467 patients; 6442 encounters) with notes from encounters up to 7 days post-event	NLP concept/term matching with combinations of concepts, including “rule-out” concepts, to infer overdose and intent	Intentional overdose (Inten-OD): Sensitivity 0.97; Specificity 0.94 (also reported: Unintentional OD Sens 0.79, Spec 0.85; PPV Uninten/Inten 0.86/0.87)	Enables automated opioid overdose surveillance with intent subtype classification (suicide attempt vs. unintentional) from EHR text.
León et al., 2025 [47]	Opioid-related neurocognitive symptoms (NCSs)	VHA retrospective cohort; 55,652 patients prescribed long-term opioid therapy (LTOT) in 2018; clinical notes assessed pre-, during-, and post-LOT	NLP-based information extraction from clinical notes	3.1% had opioid-related NCSs; highest incidence during LOT (active therapy)	Enables EHR-scale surveillance/monitoring of opioid-related neurocognitive harms during long-term therapy
Ogilvie et al., 2021 [48]	Pediatric fentanyl overdose/poisoning in children ≤ 6 years (incl. possible TIRF involvement	Optum EHR database (1 November 2015–30 September 2019); 2,527,160 children; 39 potential cases	Diagnosis codes + NLP term combinations in notes + clinician adjudication (2 adjudicators)	Confirmed 12/39 (30.8%); false positives 25/39 (64.1%); indeterminate 2/39 (5.1%); none involved TIRF	EHR-scale rare-event surveillance; findings suggest pediatric TIRF exposure/poisoning is rare and inform/reflect adequacy of TIRF REMS.
Sinha et al., 2017 [49]	Prescription opioid dependence; demographic patterns (age/race/ethnicity/geography)	Western New York (WNY) primary care clinics (Allscripts EHR → OMOP/OHDSI); 2010–2015; 212,343 patient records	High-Throughput Phenotyping NLP (HTP-NLP) web-service with ontology matching (e.g., SNOMED CT) and assertion status; queries over problem list/diagnosis codes/med lists	Opioid dependence prevalence 0.64% over 5 years (1356/212,343; 95% CI 0.61–0.67%); highest counts in ages 29–38	Demonstrates feasibility of regional EHR + ontology/NLP infrastructure for surveillance and targeting interventions to vulnerable subgroups.
Sung et al., 2025 [50]	Neurocognitive symptoms (NCSs) and opioid overdose association among older adults initiating long-term opioid therapy (LTOT)	Veterans Health Administration (VHA) national retrospective cohort; 29,302 older adults initiating LTOT (2018 initiation window)	NLP + ICD-10 surveillance to identify NCSs; overdose via ICD-10; multivariable logistic regression for association	NLP NCS F1 = 0.90; 14% had ≥1 NCS; overdose prevalence 4.10% vs. 0.44% (NCS vs. no NCS); 76% of overdoses occurred after NCS; adjusted association AOR 8.12 (95% CI 6.31–10.45)	NCSs may be a prodromal predictor/warning sign for overdose risk in older adults on LTOT, potentially prompting clinical action (e.g., dose lowering)
Tsai et al., 2024 [51]	Develop an in silico Addiction Behavior Checklist (ABC) score from clinical notes to phenotype problematic opioid use/OUD-related behavior for SNP-heritability estimation and GWAS.	Vanderbilt University Medical Center (VUMC) BioVU biorepository; 63,207 individuals with EHR + genotype data (European *n* = 52,930; African *n* = 10,277).	NLP-derived ABC in silico scores from clinical notes; estimate SNP-based heritability and conduct GWAS (plus polygenic score associations described in Results).	SNP-heritability 4.1 ± 0.5% (European) and 10.3 ± 2.8% (African); GWAS identified a significant locus in European cohort (rs4364183; P = 2.11 × 10^−8^) in SATB1-AS1; no genome-wide significant hits in African cohort.	Demonstrates feasibility of secondary use of text-derived clinical checklist scores as scalable phenotypes for genetic discovery in opioid-related risk.
Wang et al., 2009 [52]	Drug–ADE signal/association detection (pharmacovigilance)	New York Presbyterian Hospital (NYPH); 25,074 inpatient discharge summaries (2004)	MedLEE NLP extraction (UMLS-coded drug & ADE concepts) + co-occurrence association statistics (volume-test-adjusted)	Overall (known ADEs): Recall 0.75; Precision 0.31	Feasibility framework for active pharmacovigilance using unstructured EHR narrative text
Zhu et al., 2022 [53]	Identify opioid use disorder (OUD) from clinical notes in adult non-cancer patients on chronic opioid therapy (COT); compare NLP vs. ICD-coded OUD	MUSC EHR/Research Data Warehouse; 13,654 adult non-cancer COT patients (2013–2018); 75/25 train/test split	Linguamatics I2E (v5.4) rule-based NLP (lexicon + negation handling + query modules) with manual chart review gold standard	Precision 98.5%, Recall 100%, F-measure/F1 99.2% (document-level); NLP vs. ICD concordance κ = 0.63	High-accuracy OUD ascertainment from notes; 33.3% of NLP-identified OUD cases lacked ICD-coded OUD, supporting NLP + ICD combined identification
Vorontsova et al., 2021 [54]	OUD case ascertainment (develop NLP to identify OUD vs. structured ICD search)	Indiana University + Regenstrief Institute; opioid orders from Indiana University and Eskenazi Health Systems (2009–2015); full cohort 532,796 patients; 300-note corpus for dictionary refinement	Dictionary-based NLP “machine”; refined OUD dictionary/ontology with removal of patient instructions and addition of negation	Recall improved 58% → 91% after refinement; NLP identified 24,769 additional OUD cases beyond ICD-coded cases	Markedly improves OUD identification/case ascertainment compared with structured ICD-only search at population scale
Palmer et al., 2015 [55]	Prevalence of documented problem opioid use (overuse/misuse/abuse) in chronic opioid therapy (COT) patients	Group Health clinics; 22,142 adult COT patients (2006–2012)	NLP identification of candidate notes + computer-assisted manual review	7-year prevalence of documented problem opioid use: 9.4%	Efficient, scalable surveillance of clinically recognized problem opioid use in EHR notes
Singleton et al., 2022 [56]	Compare CVD prevalence among patients with OUD identified by NLP vs. ICD-10-CM codes	University of Kentucky (Lexington, KY); anonymized EHR from inpatient & emergency department visits (2019); *N* = 22,701 patients	Rule-based NLP on clinical notes to identify OUD vs. ICD-10-CM OUD codes (CVD ascertained via ICD codes)	NLP identified 395 OUD patients missed by ICD-10-CM (395/1878 = 21% of OUD cases)	Reduces under-ascertainment of OUD and CVD burden (e.g., heart failure, ischemic heart disease) and may prevent overestimation of infective endocarditis prevalence when using ICD codes alone

This synthesis focuses on the foundational role of rule-based systems in providing interpretable and transparent case-finding for overdose, opioid use disorder, and aberrant drug-related behaviors across diverse clinical settings. Abbreviations: *ABC: Addiction Behaviors Checklist; ADE: Adverse Drug Event; ADRB: Aberrant Drug-Related Behavior; AOR: Adjusted Odds Ratio; AUC: Area Under the Curve; BioVU: Vanderbilt University’s Biorepository; CI: Confidence Interval; COT: Chronic Opioid Therapy; CUI: Concept Unique Identifier; CVD: Cardiovascular Disease; DIRE: Diagnosis, Intractability, Risk, Efficacy; ED: Emergency Department; EHR: Electronic Health Record; EMS: Emergency Medical Services; F1: F1-score; GWAS: Genome-Wide Association Study; HTP: High-Throughput Phenotyping; ICD-10-CM: International Classification of Diseases, 10th Revision, Clinical Modification; κ: Cohen’s kappa; KPNW: Kaiser Permanente Northwest; KPW: Kaiser Permanente Washington; LTOT: Long-Term Opioid Therapy; NCS: Neurocognitive Symptoms; NHCS: National Hospital Care Survey; NLP: Natural Language Processing; NYPH: New York Presbyterian Hospital; OHDSI: Observational Health Data Sciences and Informatics; OMOP: Observational Medical Outcomes Partnership; ORT: Opioid Risk Tool; OUD: Opioid Use Disorder; PheWAS: Phenome-Wide Association Study; PPV: Positive Predictive Value; NPV: Negative Predictive Value; RegEx: Regular Expressions; REMS: Risk Evaluation and Mitigation Strategy; Sens: Sensitivity; Spec: Specificity; SNOMED CT: Systematized Nomenclature of Medicine Clinical Terms; SOAPP: Screener and Opioid Assessment for Patients with Pain; SNP: Single Nucleotide Polymorphism; TF-IDF: Term Frequency–Inverse Document Frequency; TIRF: Transmucosal Immediate-Release Fentanyl; UMLS: Unified Medical Language System; VHA: Veterans Health Administration; VUMC: Vanderbilt University Medical Center; WNY: Western New York.*

**Table 3:** Summary of Machine Learning and Deep Learning (ML/DL) Studies for Opioid Safety Surveillance (*n* = 18).

Study	Objective/Phenotype	Setting & Data	Model/Method	Key Performance Metrics	Clinical Advancement
Afshar et al., 2023 [57]	Real-time opioid misuse screening (risk for OUD) delivered via BPA	Hospitalized adults (≥18 years); silent test *n* = 100 encounters (2021), oversampled for substance-use ICD codes	SMART-AI (CNN) using cTAKES-extracted UMLS CUIs from EHR notes	Sens 93% (95% CI 66–99), Spec 92% (95% CI 84–96); External/temporal validation (prior study): AUPRC 0.87 (95% CI 0.84–0.91)	End-to-end, real-time NLP + DL CDS pipeline returning an Epic BPA within minutes; reproducible protocol/pseudocode for deployment.
Afshar et al., 2019 [58]	Identify EHR-based subtypes of opioid misuse among hospitalized (ED/inpatient) adults (prognostic enrichment).	Urban tertiary academic center (Loyola University); 2007–2017; 6224 opioid-misuse encounters; 422,147 clinical notes.	Latent class analysis (LCA) on 8 structured EHR variables + cTAKES-derived CUIs from notes with LDA topic modeling (20 topics) for face validity.	Opioid-misuse definition validation Sensitivity 88.6%, Specificity 78.5%; 4-class solution with reported class separation (posterior probabilities); outcome stratification (e.g., 30-day readmission 13.9% highest in Class 1; AMA discharge 12.3% highest in Class 2).	Clinically interpretable risk/utilization subtypes enabling targeted interventions and care pathways (prognostic enrichment).
Afshar et al., 2021 [59]	External validation of an opioid misuse classifier	Rush University Medical Center; 56,227 adult hospitalizations (screened inpatient cohort)	CNN using UMLS Concept Unique Identifiers (CUIs) from EHR notes (cTAKES) + isotonic calibration	Calibrated model (optimal cutpoint): Sensitivity 0.81; Specificity 0.99; PPV 0.72	Calibration improves PPV (precision) and supports real-world inpatient triage by reducing false alerts
Afshar et al., 2022 [60]	Multisubstance misuse screening (alcohol, opioid, non-opioid drugs)	Hospitalized adults: Rush University (*n* = 54,915, training/temporal validation) and Loyola University (*n* = 1991, external validation); first 24 h EHR notes	Multilabel CNN using UMLS CUIs	Temporal validation: overall AUROC 0.97; External validation: opioid misuse AUPRC 0.91, AUROC 0.94	Enables single-model, hospital-wide automated screening for alcohol and drug misuse to support referral triage
Taylor et al., 2023 [61]	Computational phenotypes of opioid-related ED presentations derived from clinical notes + structured EHR	Regional healthcare network (10 EDs, 2013–2020); adults ≥ 18 with prior/current opioid-related ICD-10 diagnosis; 82,577 ED visits	MedCAT clinical concept extraction → UMLS CUIs (+structured EHR features) → LDA topic modeling (30 topics) → K-means clustering on topic embeddings	9 clusters; one-year survival 84.2–96.8%; one-year ED returns ~9–34%; one-year opioid events 10–17%; MOUD 17–43%; median CCI 2–8	Identifies clinically meaningful opioid-related ED subgroups beyond ICD labels to support targeted interventions and resource allocation (e.g., high-mortality vs. high-utilization cohorts)
Blackley et al., 2020 [62]	Inpatient OUD identification (screening) from free-text notes to facilitate addiction-service referral	Brigham & Women’s Hospital (Mass General Brigham); 22,626 admissions/patients; 846,302 notes (ED notes, inpatient progress notes, prior discharge summaries)	Rule-based NLP (MTERMS) + ML classifiers (LR, SVM, KNN, RF); also evaluated a DNN	Best validation F1 = 0.9683 (SVM/KNN; precision 0.9730, recall 0.9722; *n* = 216 validation set)	Demonstrates feasibility for near-real-time inpatient OUD screening to trigger/streamline addiction-service consults (future EHR integration).
Green et al., 2019 [63]	Opioid-related overdose (OOD) detection and classification (e.g., heroin involvement; suicide/suicide attempt; substance abuse involvement)	Kaiser Permanente Northwest (KPNW), 2008–2014; EHR/claims + chart review; validation dataset *n* = 1136 chart-audited events (suspected OOD + at-risk noncases); portability assessed in KPWA, Optum, and TennCare	Code-based ICD algorithms; LASSO-assisted feature selection with logistic regression for some classifications; NLP-enhanced variants (clinical-text NLP-derived variables) evaluated for suicide/attempt and abuse involvement	OOD detection F1 = 0.92 (validation); suicide/suicide attempt F1 = 0.80 (NLP-enhanced) (code-based F1 = 0.74); portability OOD F-scores ≥ 0.92 across sites	Validated and assessed portability of overdose detection and key subtype/intent classifiers across multiple, heterogeneous health systems and data environments.
Jeffery et al., 2024 [64]	Noisy-label/weak-supervision approach to identify opioid-induced respiratory depression (OIRD)	Vanderbilt University Medical Center (Synthetic Derivative EHR); 52,861 post-operative visits	Snorkel expert-informed labeling functions (LFs) → Generative model (probabilistic labels) → weighted Random Forest Discriminative classifier	Hold-out manually adjudicated Test Set (*n* = 599; prevalence 0.83%): Sensitivity 1.0, AUC 0.988, F1 0.417 (Accuracy 0.977)	Demonstrates scalable identification of a rare, hard-to-ascertain harm; high sensitivity supports reducing manual review by prioritizing high-probability records.
Lingeman et al., 2017 [65]	Opioid-related aberrant behavior in outpatient primary care notes	UMass Memorial Health Care; 112 annotated outpatient primary care notes (44 positive)	SVM with hand-crafted features + sentiment features (10-fold stratified cross-validation)	Best accuracy 81.4% (SD 0.12; 10-fold CV)	Text-only surveillance of opioid-related aberrant behavior without relying on structured billing-code labels (for classification).
Poulsen et al., 2022 [66]	OUD-related annotation schema and sentence-level classification of OUD context in discharge summaries	MIMIC-III critical care (ICU) discharge summaries; 100 patients annotated; train 2127 sentences (66 pts), test 1143 sentences (29 pts)	Feature-engineered logistic regression baseline vs. AutoGluon TextPredictor (ELECTRA transfer learning)	AutoGluon (test): Opioid type F1 = 99; Drug screening F1 = 94	Schema-driven extraction enabling scalable characterization of OUD context from clinical text (best automation for opioid type and drug screening)
Sharma et al., 2020 [67]	Compare PHI-free (CUI/character) vs. PHI-laden (word n-gram) models for opioid misuse classification	Loyola University Medical Center inpatient EHR notes (2007–2017); case–control annotated dataset *n* = 1000	Neural models (CNN, Deep Averaging, Max Pooling, Deep Averaging + Max Pooling) and logistic regression using CUI/character (PHI-free) vs. word n-gram (PHI-laden) features	Best test AUROC 0.94 (PHI-laden CNN-Word; PHI-free DeepAvg + MaxPool-CUI); Also reports F1/precision/recall/specificity/NPV	Releases public, PHI-free opioid-misuse classifiers (CUI-based) to enable safer sharing/deployment without PHI leakage concerns
Sharp et al., 2025 [68]	Predict opioid overdose-related death within 12 months after an ED visit	Urban safety-net ED EHR (2011–2018) linked to state mortality records (2012–2019); *n* = 5656 (729 decedents; 4927 controls)	QuickUMLS extraction of UMLS CUIs from clinical notes + structured EHR features; mutual information feature selection (top 50); ML classifiers (XGBoost [tuned], Random Forest, Logistic Regression)	Test set (cutoff > 0.5): XGBoost AUC-ROC 0.92, F1 0.65 (also AUC-PR 0.72; precision 0.74; recall 0.58)	Demonstrates feasibility of EHR note-augmented ML to flag ED patients at high risk for fatal opioid overdose, supporting prospective clinical decision support and ED-initiated prevention/treatment workflows
Thompson et al., 2021 [69]	Fairness/bias audit of misuse classifier	Loyola development/internal dataset (*n* = 1000) + Rush external validation cohort of screened adult inpatient encounters (*n* = 53,974; 2017–2019)	CNN opioid misuse classifier; bias audit using subgroup error metrics (bootstrapped CIs) + LIME feature inspection; post hoc mitigation via subgroup threshold adjustment and isotonic recalibration	Unmitigated Black FNR 0.32 vs. White FNR 0.17; after subgroup recalibration, Black FNR 0.24 vs. White FNR 0.21 (bias removed)	Shows that routine bias audits can reveal clinically important subgroup harms and that simple post hoc recalibration can mitigate racial gaps with minimal tradeoffs.
Yin et al., 2025 [70]	Predict/identify OUD within 12 months (calendar year) and examine how dual-system use and patient factors interact to influence OUD risk (OUD defined by ICD-9/10 + NLP from notes)	Washington, DC & Baltimore VA Medical Centers (2012–2019); VA CDW data in VINCI; 222,370 patients contributing 856,299 patient-year instances	ResNet-style DNN (residual blocks) for OUD prediction + explainable AI impact/interaction scoring; outcome ascertainment includes NLP classifier (SVM + regular expressions) on note snippets plus ICD codes; logistic regression benchmark; class imbalance handled via random under-sampling in training	Test: AUC 0.784, F1 0.462 (also accuracy 0.728; precision 0.35; recall 0.68)	Confirms dual-system use is associated with higher OUD risk (and interacts with age), helping identify veteran subgroups who may warrant targeted attention/interventions
Badger et al., 2019 [71]	Opioid overdose severity phenotyping/classification (false positive/mild/moderate/severe)	Marshfield Clinic Health System; 298 chart-reviewed labeled overdose events (screened via ICD-9/10 codes)	Random forest using OMOP CDM features + NLP (cTAKES-derived UMLS CUIs)	Micro-averaged AUC (all classes) 0.893; Severe-class AUC 0.982	Introduces severity stratification enabling higher-granularity overdose phenotypes beyond ICD codes
Carrell et al., 2017 [72]	Identify problem prescription opioid use (POU) (clinician-labeled misuse/abuse/addiction or death) among chronic opioid therapy patients	Group Health EHR (2006–2012); *N* = 15,498 chronic opioid therapy (≥70 days’ supply/90 days); train/validation *N* = 7749 each; labels from validated NLP-assisted manual review	Two-model algorithm: logistic regression using 6 EHR-derived predictors + machine-learned model using NLP-extracted note features; POU+ if either model exceeds cut-point (OR ensemble)	Validation: Sensitivity 56%, Precision 76% (POU prevalence 9.4%)	Fully automated EHR + text algorithm enabling population-scale surveillance/epidemiologic research (modest performance noted).
Sorbello et al., 2023 [73]	SPINEL prototype to identify opioid-related ADE (ORADE) safety signals from free-text discharge summaries for pharmacovigilance/regulatory use	MIMIC-III discharge summaries; 227 ICD-9–prescreened summaries (reference set; 181 train/46 test)	Hybrid NLP pipeline: keyword + trigger-phrase rules (and naloxone signal) + MedSpaCy sectionizer/context + Spark NLP for Healthcare NER (biLSTM + CNN) with concept mapping (UMLS/MedDRA) and dashboard visualization	Accuracy 0.66; Recall 0.69; Precision 0.64; F1 0.67 (test set of 46 summaries)	Web-based dashboard that visualizes opioid safety signals and supports FDA/CDER regulatory review, reducing manual chart-review burden; favorable usability feedback from FDA staff
Workman et al., 2024 [74]	Identify problematic opioid use from clinical notes (all settings) and compare NLP-only vs. ICD-coded OUD patients	U.S. Veterans Affairs (Baltimore, MD + Washington, DC stations), 2012–2019; *n* = 222,371 patients; 81.13 M total notes; 3.52 M notes containing key phrases	Hybrid NLP: 145 regular expressions with sequential voting (rule-based) + SVM snippet classifier Unseen test set (*n* = 161 snippets)	Sensitivity/Recall 88.4%, Specificity 96.6%, Precision/PPV 90.4%, Accuracy 94.4%; Identifies many more patients than ICD codes alone (NLP-only 57,331 vs. All-ICD 6997)	Recovers problematic use missed by ICD codes; tool released as open-source to support large-scale surveillance.

This table synthesizes empirical research utilizing supervised and unsupervised machine learning architectures to identify, subtype, and predict opioid-related harms from clinical text. It highlights the transition from traditional classifiers to deep learning architectures integrated directly into real-time clinical workflows. *Abbreviations: AMA: Against Medical Advice; ADE: Adverse Drug Event; AUC: Area Under the Curve; AUPRC: Area Under Precision–Recall Curve; AUROC: Area Under Receiver Operating Characteristic Curve; biLSTM: Bidirectional Long Short-Term Memory; BPA: Best Practice Alert; CDS: Clinical Decision Support; CDER: Center for Drug Evaluation and Research; CDM: Common Data Model; CDW: Corporate Data Warehouse; CI: Confidence Interval; CNN: Convolutional Neural Network; cTAKES: clinical Text Analysis and Knowledge Extraction System; CCI: Charlson Comorbidity Index; CUI: Concept Unique Identifier (UMLS); DL: Deep Learning; DNN: Deep Neural Network; ED: Emergency Department; EHR: Electronic Health Record; F1: F1-score; FDA: U.S. Food and Drug Administration; FNR: False Negative Rate; ICD: International Classification of Diseases; ICD-9/10: International Classification of Diseases, 9th/10th Revision; ICU: Intensive Care Unit; KNN: k-Nearest Neighbors; KPNW: Kaiser Permanente Northwest; KPWA: Kaiser Permanente Washington; LASSO: Least Absolute Shrinkage and Selection Operator; LCA: Latent Class Analysis; LDA: Latent Dirichlet Allocation; LF: Labeling Function; LR: Logistic Regression; MedCAT: Medical Concept Annotation Toolkit; MedDRA: Medical Dictionary for Regulatory Activities; MIMIC: Medical Information Mart for Intensive Care; ML: Machine Learning; MOUD: Medications for Opioid Use Disorder; MTERMS: Medical Term Recognition System; NER: Named Entity Recognition; NLP: Natural Language Processing; NPV: Negative Predictive Value; OIRD: Opioid-Induced Respiratory Depression; OMOP: Observational Medical Outcomes Partnership; OOD: Opioid Overdose Detection; OUD: Opioid Use Disorder; PHI: Protected Health Information; POU: Problem Opioid Use; PPV: Positive Predictive Value; QuickUMLS: QuickUMLS concept-matching tool; RF: Random Forest; SD: Standard Deviation; Sens: Sensitivity; SMART-AI: Substance Misuse Analytics & Real-time Tracking AI; Spec: Specificity; SPINEL: Safety Pharmacovigilance and Intelligence using Natural Language; SVM: Support Vector Machine; UMLS: Unified Medical Language System; VA: Veterans Affairs; VINCI: VA Informatics and Computing Infrastructure; XGBoost: Extreme Gradient Boosting; CV: Cross-Validation.*

**Table 4:** Summary of Transformer and Large Language Model (LLM) Studies for Opioid Safety Phenotyping (*n* = 10).

Study	Objective/Phenotype	Setting & Data	Model/Method	Key Performance Metrics	Clinical Advancement
Afshar et al., 2025 [75]	EHR-embedded AI screening for risk of OUD/unhealthy opioid use; evaluate implementation (non-inferiority vs. usual care)	UW Health University Hospital (Epic EHR); pre–post study of 51,760 adult hospitalizations; real-time inpatient notes with ED notes incorporated after optimization; prior external validation cohort *n* = 1991 referenced for thresholding	CNN (12.5 M params) on UMLS concept unique identifiers (CUIs) from notes; triggers Epic best practice alert (BPA) when score > 0.05 in first 24 h; integrated gradients for feature attribution; open-source LLM testing showed no performance gain	Implementation outcomes: completed addiction medicine consults 1.35% (pre) vs. 1.51% (post) (non-inferior); among consulted patients 30-day readmission aOR 0.53 (0.30–0.91). Prior external validation at threshold 0.05: Sens 0.87 (0.84–0.90), PPV 0.76 (0.72–0.88), NNE 1.4	Hospital-wide deployment of an EHR-integrated OUD risk screener with BPA workflow; maintained consult completion while reducing readmissions and demonstrated cost-effectiveness (ICER $6801 per readmission avoided)
Funnell et al., 2025 [76]	Drug involvement in overdose deaths from free-text ME/coroner narratives (multi-label; polysubstance)	U.S. ME/coroner death records. Internal (2020): *n* = 35,433. External validation (2023–2024): *n* = 3335 (10 counties).	Multi-label text classification comparing classic ML baselines, fine-tuned encoder models (BERT, BioClinicalBERT), and decoder-only LLMs (Qwen 3, Llama 3) with 0/3/5/10-shot prompting plus supervised fine-tuning.	Internal (best = fine-tuned BioClinicalBERT): macro-F1 0.998; AUROC 1.00. External (best LLM = Qwen-3 1.7 B, 3-shot): macro-F1 0.968 (0.961–0.974); accuracy 0.958 (0.952–0.964). External (BioClinicalBERT): macro-F1 0.966. External (Llama-3.2, 0-shot): macro-F1 0.959.	Enables scalable, faster polysubstance overdose surveillance from death narratives, reducing delays from manual ICD-10 coding.
Harel-Canada et al., 2025 [77]	Multi-label sentence-level detection of substance use in clinical text (heroin, cocaine, methamphetamine, illicit prescription opioid misuse, illicit benzodiazepine use, cannabis, injection drug use, any drug use)	MIMIC-III/IV discharge summaries (high-acuity inpatient notes); DrugDetection benchmark 8053 labeled sentences (Train 804/Val 806/Test 6443)	Compared BERT encoders and LLMs under zero-shot/few-shot in-context learning and few-shot fine-tuning; best model Llama-DrugDetector-70 B (fine-tuned)	Held-out test (*n* = 6443): F1 0.917 (0.905–0.928), Accuracy 0.926 (0.920–0.932), Sensitivity/Recall 0.959 (0.947–0.968); Rx opioid misuse F1 0.815 (0.784–0.848)	Released a public DrugDetection benchmark + open model enabling scalable substance use surveillance from hospital notes (strong performance even on challenging Rx opioid misuse)
Hosseini et al., 2025 [78]	Opioid-induced respiratory depression (OIRD)/oversedation risk prediction (binary)	UC Davis Medical Center (EMR/EPIC); adult inpatient hospitalizations receiving opioids (2010–2020); 2663 hospitalizations extracted; refined dataset 1914 after preprocessing	Fine-tuned BioBERT, ClinicalBERT, and GatorTron (GatorTronS/Base); GPT-4 (ChatGPT) used for feature selection (52 → 25 features); baselines: SVM, Random Forest, XGBoost	LLMs (validation accuracy): with GPT-4 feature selection—BioBERT 84.04% (vs. 79.67%), ClinicalBERT 82.02% (vs. 71.11%), GatorTron 82.22% (vs. 78.79%); Traditional ML baseline: SVM AUC 0.85 (accuracy 74.36%)	Shows feasibility of predicting OIRD/oversedation risk from EMR-derived narratives; GPT-4-based feature selection improves LLM predictive accuracy over using all features and over traditional ML baselines
Kwon et al., 2024 [79]	Opioid-related aberrant behaviors (ORABs): Confirmed Aberrant Behavior (CAB) and Suggested Aberrant Behavior (SAB) (ODD has 9 multi-label categories total)	MIMIC-IV de-identified EHR notes; 750 notes annotated (500 patients) → 399 notes (325 patients) with current opioid prescriptions; 3718 labeled instances	BioBERT and BioClinicalBERT; compared standard fine-tuning vs. prompt-based fine-tuning; optional LLM augmentation via Flan-T5 XL paraphrasing	Best baseline (BioClinicalBERT, prompt-based): Macro AUPRC 88.17; Macro F1 82.86 (macro over 9 labels). CAB (prompt-based BioClinicalBERT): AUPRC 90.52; F1 78.25. CAB + Flan-T5 XL augmentation: AUPRC 93.86; F1 87.36 (CAB only).	Public, expert-annotated benchmark dataset for ORAB detection from EHR notes, enabling comparable evaluation of ORAB extraction models.
Mitra et al., 2021 [80]	SBDH extraction from ICU EHR notes; risk factors for nonfatal opioid overdose (ICD-9-defined) leading to ICU admission	MIMIC-III ICU (Beth Israel Deaconess MC; 2001–2012); *n* = 48,869 admissions (37,361 patients); SBDH NLP trained on 1000 annotated notes (relevant sections from discharge summaries/social work/rehab notes)	medSpaCy section extraction + BERT-based SBDH extraction (word-level); sequential forward selection; multivariable logistic regression association analysis	Adjusted odds ratios (aOR, 95% CI): Drug use disorder 8.17 (5.44–12.27); Bipolar disorder 2.69 (1.68–4.29); Current illicit drug use 2.06 (1.20–3.55); NLP evaluation metrics not reported in main text (in appendix), but NLP captured 99.82% of SBDH vs. 0.18% via ICD codes	Demonstrates scalable NLP extraction of SBDH from ICU notes and integration into overdose risk characterization (supports incorporating NLP-derived SBDH into OD risk assessment)
Paredes et al., 2025 [81]	Extract opioid overdose, OUD, and related information (including naloxone/Narcan revive medication/procedures, problematic prescription opioid use, opioid urine test results, opioid substances, and status) from clinical narratives (9 concept categories).	UF Health Integrated Data Repository (IDR; >300 M clinical notes). Keyword-filtered sample with 600 annotated clinical notes; split 420 train/60 dev/120 test.	Encoder-based LLMs (BERT, Bio + ClinicalBERT, GatorTron-base) fine-tuned for NER (BIO tagging). Decoder-based generative LLM (GatorTronGPT) formulated as QA and optimized via p-tuning (soft prompts; NeMo pipeline). Evaluated with strict vs. lenient entity matching.	Overall (micro-averaged) on 120-note test set: GatorTronGPT strict P/R/F1 = 0.8565/0.8710/0.8637; lenient F1 = 0.9057. GatorTron-base strict/lenient F1 = 0.8461/0.8580.	Provides a systematic NLP tool to extract overdose/OUD-related entities for a computable patient profile to support opioid-related research and potential EHR-enabled identification of high-risk patients.
Shahid et al., 2024 [82]	ED encountering opioid misuse detection (manual annotation; NIDA/NSDUH-consistent definition)	UIHealth ED (Epic EHR); *n* = 1123 encounters; temporal split test *n* = 169	SMART-AI (CNN over cTAKES-derived UMLS CUIs), end-to-end fine-tuned for ED domain adaptation; comparators: LR, SVM, decision tree, XGBoost; BERT/BioBERT/Longformer variants; ICD-10-CM baseline	Best overall (highest AU_PRC) SMART-AI fine-tuned: AU_PRC 0.9476; AU_ROC 0.9464; F1 0.8507; Precision 0.9661. XGBoost (CUIs) AU_PRC 0.936. ICD-10-CM baseline F1 0.8308.	NLP-based ED screening outperforms ICD-10-CM code-based detection for opioid misuse.
Kashyap et al., 2023 [83]	Predict (1) opioid prescription during admission and (2) OUD diagnosis (ICD-9-defined) from EHR data	MIMIC-III ICU; 53,423 admissions. Balanced test: Opioid Rx 1895/1895 (total 3790); OUD 76/76 (total 152). Unbalanced test: Opioid Rx 2709/798 (total 3507); OUD 40/5037 (total 5077)	Multimodal deep learning: FFN for structured static features + Transformer for time-varying events + hierarchical Transformer over notes with ClinicalBERT embeddings; concatenation + FFN classifier	Balanced test (mean ± SD over 10 runs): Opioid Rx F1 0.88 ± 0.003, AUROC 0.93 ± 0.002; OUD F1 0.82 ± 0.05, AUROC 0.94 ± 0.008. Calibration (isotonic): Brier 0.102 (Rx), 0.146 (OUD)	Proof-of-concept multimodal ICU risk prediction using structured + clinical text; proposes point-of-care potential (no external validation)
Lenert et al., 2022 [84]	Adapt ACT into the Opioid Overdose Network (O2-Net) to support ED opioid-overdose cohort identification (tiered ICD-based e-phenotypes), enrich cases via sentence-level NLP (naloxone response, intentionality), and deploy an ED Opioid Smart Tool (OST) documentation template/reminder to improve capture and promote take-home naloxone.	O2-Net consortium (MUSC, Dartmouth, University of Kentucky, UCSD) on federated i2b2/SHRINE. NLP pipeline: NER + Rules identified 1513 candidate opioid-related overdose-content sentences from a random 20% sample of 2.47 M notes (manual review used for evaluation). OST evaluation at MUSC: high-probability cases *n* = 389 (Charleston) and *n* = 292 (regional hospitals).	CLAMP-based NLP with 4 approaches: NER + Rules; SVM (uni/bi/tri-grams); BERT classifier; BERT + post-processing/inference rules (negation/hypothetical/non-patient subject filtering). OST template + reminder triggered by chief-complaint terms (“Drug Overdose,” “naloxone,” “Narcan”).	Best NLP (BERT + rules): Precision 0.97, Recall 0.90, F1/F score 0.94. OST reminder triggered: 21% (81/389) Charleston; 45% (130/292) regional hospitals. OST template used (among triggered): 23/81 (28%) Charleston; 18/130 (14%) regional hospitals. Take-home naloxone Rx when template used: 66% vs. 16% at MUSC (*p* < 0.0001); 44% vs. 16% at community hospitals (*p* < 0.0001).	Demonstrates feasibility of a task-focused federated network (O2-Net) and portable NLP deployment; OST template/reminder in live EHR workflow was associated with substantially higher take-home naloxone prescribing, motivating further effectiveness study within the network.

This table summarizes studies utilizing transformer-based architectures and large language models for identifying opioid-related safety signals from clinical text. Studies are organized by publication year. Performance metrics are reported as point estimates with 95% confidence intervals (CI) where available. Abbreviations: *aOR = adjusted odds ratio; AUC = area under the curve; AUROC = area under the receiver operating characteristic curve; AUPRC = area under the precision–recall curve; BERT = Bidirectional Encoder Representations from Transformers; BioBERT = BERT pre-trained on biomedical literature; BioClinicalBERT = BERT pre-trained on clinical notes; BPA = Best Practice Alert; CI = confidence interval; CLAMP = Clinical Language Annotation, Modeling, and Processing; CNN = convolutional neural network; CUI = Concept Unique Identifier; ED = emergency department; EHR = electronic health record; EMR = electronic medical record; Epic = Epic Systems electronic health record; F1 = F1-score (harmonic mean of precision and recall); FFN = feed-forward network; GPT = Generative Pre-trained Transformer; ICU = intensive care unit; IDR = Integrated Data Repository; LLM = large language model; LR = logistic regression; ME = medical examiner; MIMIC = Medical Information Mart for Intensive Care; ML = machine learning; MUSC = Medical University of South Carolina; NER = named entity recognition; NLP = natural language processing; NNE = number needed to evaluate; O2-Net = Opioid Overdose Network; OIRD = opioid-induced respiratory depression; OUD = opioid use disorder; P/R = precision/recall; PPV = positive predictive value; QA = question answering; Rx = prescription; SBDH = social and behavioral determinants of health; SVM = support vector machine; UCSD = University of California San Diego; UF = University of Florida; UMLS = Unified Medical Language System; UW = University of Wisconsin; XGBoost = extreme gradient boosting; ACT = Accrual to Clinical Trials; BIO = Begin–Inside–Outside; Brier = Brier score; CAB = Confirmed Aberrant Behavior; ICER = Incremental Cost-Effectiveness Ratio; i2b2 = Informatics for Integrating Biology and the Bedside; NeMo = NVIDIA NeMo; NIDA = National Institute on Drug Abuse; NSDUH = National Survey on Drug Use and Health; ODD = ORAB Detection Dataset; ORAB = Opioid-Related Aberrant Behavior; OST = Opioid Smart Tool; p-tuning = prompt tuning; SAB = Suggested Aberrant Behavior; SHRINE = Shared Health Research Information Network.*

## Data Availability

No new data have been generated in this study. Topics discussed are extracted from the articles cited in the manuscript; as a result, data sharing is not applicable to this study.

## Supplementary Materials

The following supporting information can be downloaded at: https://www.mdpi.com/article/10.3390/jcm15041649/s1. Table S1: Glossary of abbreviations and acronyms terms, as well as their expansion, Table S2: Glossary of key technical concepts used in this review.
