# Supplementary material for: Artificial Intelligence for Opioid Safety Surveillance from Clinical Text: A Clinically Focused Review
Source: J Clin Med. 2026 Feb 22;15(4):1649. doi: 10.3390/jcm15041649 (PMC12942020; doi:10.3390/jcm15041649)
Supplement: Supplementary file 1 [file jcm-15-01649-s001.zip › jcm-4130995-supplementary.pdf]

Supplementary Table S1. Glossary of abbreviations and acronyms terms, as well as their expansion. Abbreviations appearing in the main text, tables, and figures are listed (A-Z) with their full expansions and a brief description of how each term is used in the context of clinical text–based opioid safety phenotyping, evaluation, and implementation. Where multiple variants exist (e.g., AUROC/AUC-ROC), the preferred form is listed with common aliases noted.

Term	Explanation	Term	Explanation
ABC	Addiction Behavior Checklist: Risk-screening checklist referenced in included studies.	ACT	Accrual to Clinical Trials: Variable used in an included study; in the manuscript's abbreviations list.
ADE	Adverse Drug Event: Harmful event related to medication exposure; often used interchangeably with adverse event in this review.	ADRB	Aberrant Drug-Related Behavior: Behaviors suggesting nonmedical use/misuse; used in opioid-misuse phenotyping.
AI	Artificial Intelligence: Umbrella term covering ML/DL/LLM approaches.	AMA	Against Medical Advice: Discharge from care against clinician advice; appears in cohort/phenotype descriptions.
AOR	Adjusted Odds Ratio: Odds ratio adjusted for covariates.	AUC	Area Under the Curve: Generic AUC; in this manuscript usually for ROC/PR curves.
AUPRC	Area Under the Precision–Recall Curve: Area under the precision–recall curve; primary discrimination metric for imbalanced outcomes. (Also written as AUC-PR.)	AUROC	Area Under the ROC Curve: Area under the receiver operating characteristic curve; discrimination metric for binary classification regardless of prevalence. (Also written as AUC-ROC.)
BERT	Bidirectional Encoder Representations from Transformers: Transformer encoder model family used for clinical NLP tasks.	BIO	Begin–Inside–Outside: Sequence-labeling scheme for NER tagging.
BioBERT	Biomedical BERT: BERT variant pretrained on biomedical corpora; used for clinical/biomedical NLP baselines.	BPA	Best Practice Alert: EHR-based clinical decision support alert (e.g., an Epic Best Practice Alert) used for real-time workflow integration.
Brier score	Brier score: Proper scoring rule measuring accuracy of probabilistic predictions; lower is	CAB	Confirmed Aberrant Behavior: Confirmed aberrant behavior: explicit evidence of loss of control or non-

	better (often used for calibration).		prescribed use (e.g., sharing/diversion).
CCI	Charlson Comorbidity Index: Comorbidity burden score often used as a covariate.	CDC	Centers for Disease Control and Prevention: U.S. public health agency; cited for overdose statistics.
CDER	Center for Drug Evaluation and Research: FDA center; appears in pharmacovigilance context.	CDM	Common Data Model: Standardized data schema (e.g., OMOP CDM) enabling multi-site analyses.
CDS	Clinical Decision Support: Tools to support clinician decisions (alerts, reminders, triage).	CDW	Corporate Data Warehouse: Used in VA context (VA Corporate Data Warehouse).
CI	Confidence Interval: Uncertainty interval for an estimate.	CLAMP	Clinical Language Annotation, Modeling, and Processing: Clinical NLP toolkit used in included studies.
ClinicalBERT	Clinical BERT: BERT variant pretrained/fine-tuned on clinical notes; used for clinical NLP baselines.	CNN	Convolutional Neural Network: Neural network using convolutional filters; applied to text classification by learning local n-gram–like patterns over tokens/features.
COT	Chronic Opioid Therapy: Long-term opioid use; used for cohort definition.	CT	Clinical Terms: As in SNOMED CT (Clinical Terms).
cTAKES	clinical Text Analysis and Knowledge Extraction System: Apache NLP system often used for clinical concept extraction and mapping to UMLS.	CUI	Concept Unique Identifier: UMLS concept identifier extracted from text (often via cTAKES/QuickUMLS).
CV	Cross-Validation: Resampling procedure for model evaluation/tuning.	CVD	Cardiovascular Disease: Comorbidity label in included studies.
DAST	Drug Abuse Screening Test: Substance use screening instrument referenced in risk modeling.	DIRE	Diagnosis, Intractability, Risk, Efficacy: Risk tool used for chronic pain/opioid therapy stratification.
DL	Deep Learning: Neural-network based ML approaches.	ED	Emergency Department: Acute care setting; many cohorts are ED-based.
EHR	Electronic Health Record: Longitudinal	ELECTRA	Efficiently Learning an Encoder that Classifies

	clinical data system; includes structured fields and text notes.		Token Replacements Accurately: Discriminative transformer pretraining using replaced token detection (RTD); learns to detect replaced tokens (vs masked language modeling).
EMR	Electronic Medical Record: Often used interchangeably with EHR in included studies.	EMS	Emergency Medical Services: Pre-hospital care context; appears in overdose-related outcomes.
EPIC	Epic (EHR platform): Commercial EHR platform used for BPA/workflow deployment.	F1	F1-score: Harmonic mean of precision and recall.
FDA	U.S. Food and Drug Administration: Regulatory body; referenced for surveillance/regulatory review.	FFN	Feed-Forward Network: Dense neural network component used in multimodal architectures.
FN	False Negative: Missed positive case.	FNR	False Negative Rate: FN / (FN+TP); subgroup disparities discussed in bias audits.
FP	False Positive: Incorrectly predicted positive case.	GPT	Generative Pre-trained Transformer: Family of generative LLMs referenced in LLM-era studies.
GPT-4	Generative Pre-trained Transformer 4: Specific GPT generation used in some included studies (e.g., feature selection).	GWAS	Genome-Wide Association Study: Study design linking text-derived phenotypes to genetic loci.
HTP	High-Throughput Phenotyping: Automation of phenotyping at scale from EHR data.	HTP-NLP	High-Throughput Phenotyping NLP: Web-service/pipeline for scalable phenotyping from text.
I2E	Linguamatics I2E: Text-mining platform used for rule-based extraction.	ICD	International Classification of Diseases: Billing/diagnostic code system used as baseline or comparator.
ICD-10	International Classification of Diseases, 10th Revision: Current ICD version	ICD-10-CM	ICD-10 Clinical Modification: U.S. clinical modification of ICD-10 codes.

	used in many EHR cohorts.		
ICD-9	International Classification of Diseases, 9th Revision: Older ICD version used in historical cohorts.	ICER	Incremental Cost-Effectiveness Ratio: Cost-effectiveness metric reported for workflow adoption.
ICU	Intensive Care Unit: High-acuity inpatient setting used in MIMIC cohorts.	IDR	Integrated Data Repository: Institutional data repository (e.g., UF Health IDR).
KNN	k-Nearest Neighbors: Baseline supervised classifier referenced in some pipelines.	KPNW	Kaiser Permanente Northwest: Health system used in cohort/study settings.
KPW	Kaiser Permanente Washington: Health system used in cohort/study settings (also abbreviated KPWA in some sources).	LASSO	Least Absolute Shrinkage and Selection Operator: Regularized regression used for feature selection.
LCA	Latent Class Analysis: Unsupervised model used for misuse subtyping.	LDA	Latent Dirichlet Allocation: Topic model applied to NLP-derived features.
LF	Labeling Function: Heuristic labeling rule (e.g., in Snorkel pipelines).	LIME	Local Interpretable Model-agnostic Explanations: Post-hoc interpretability method.
LLM	Large Language Model: Large-scale neural language model (typically transformer-based) used to understand and/or generate text; in this review, includes LLM-based extraction and reasoning.	LOT	Line of Therapy: Treatment line variable used in some oncology-related analyses.
LR	Logistic Regression: Common baseline classifier; also used in hybrid pipelines.	LTOT	Long-Term Opioid Therapy: Long-duration opioid use; cohort definition in some studies.
ME	Medical Examiner: Medicolegal narratives from medical examiner/coroner systems (death investigation reports).	MEDLINE	MEDLINE: NLM bibliographic database used for literature search (commonly accessed via PubMed).
medSpaCy	medSpaCy: Clinical NLP toolkit built on spaCy; commonly used for section detection and	MIMIC	Medical Information Mart for Intensive Care: Open critical-care EHR dataset family.

	attribute assertion (negation/uncertainty).		
MIMIC-III	Medical Information Mart for Intensive Care III: Earlier MIMIC version; ICU-focused.	MIMIC-IV	Medical Information Mart for Intensive Care IV: Current MIMIC version; ICU/hospital data.
ML	Machine Learning: Supervised/unsupervised statistical learning methods.	MOUD	Medications for Opioid Use Disorder: e.g., buprenorphine, methadone, naltrexone.
MTERMS	Medical Term Recognition System: Rule-based NLP system used for term recognition/extraction.	MUSC	Medical University of South Carolina: Site referenced in O2-Net studies.
NCS	Neurocognitive Symptoms: Opioid-related neurocognitive symptom phenotype.	NER	Named Entity Recognition: Extraction of spans/entities from text (drugs, behaviors, events).
NHCS	National Hospital Care Survey: U.S. survey dataset used for validation/benchmarking.	NIDA	National Institute on Drug Abuse: U.S. research institute; appears in benchmarking/resources.
NLP	Natural Language Processing: Computational methods for analyzing text.	NNE	Number Needed to Evaluate: Workload metric: how many alerts/cases must be reviewed per true positive.
NPV	Negative Predictive Value: Probability that a negative prediction is truly negative.	NR	Not Reported: Used in tables when metrics are not provided.
NSDUH	National Survey on Drug Use and Health: U.S. national survey referenced for prevalence/validation.	NYP	New York-Presbyterian: Hospital system referenced in early pharmacovigilance study (also appears as NYPH).
O2	Opioid Overdose Network: As in O2-Net, a federated overdose phenotyping network.	ODD	ORAB Detection Dataset: Benchmark dataset for opioid-related aberrant behavior (ORAB) detection labels used in the review.
OHDSI	Observational Health Data Sciences and Informatics: Open-science consortium supporting OMOP and methods/tooling.	OIRD	Opioid-Induced Respiratory Depression: Serious opioid toxicity phenotype.
OMOP	Observational Medical Outcomes Partnership: Original Observational	OOD	Opioid Overdose (manuscript shorthand): Overdose phenotype

	Medical Outcomes Partnership initiative; OMOP Common Data Model is now maintained/advanced by the OHDSI community.		defined using codes and/or text.
OR	Odds Ratio: Association measure used in observational analyses.	ORAB	Opioid-Related Aberrant Behaviors: Behavior phenotype class (confirmed/suggested) used in benchmark studies.
ORADE	Opioid-Related Adverse Drug Event: Opioid-related adverse event category used in pharmacovigilance prototypes.	ORT	Opioid Risk Tool: Risk screening instrument for opioid misuse.
OST	Opioid Smart Tool: EHR documentation/decision-support tool used in O2-Net context.	OUD	Opioid Use Disorder: Problematic opioid use meeting diagnostic criteria; often under-coded in ICD.
PheWAS	Phenome-Wide Association Study: Association analysis testing links between a genetic variant and many phenotypes (often across EHR-derived outcomes).	PHI	Protected Health Information: Identifiable health information; PHI-free features used to enable safer sharing.
POU	Problematic Opioid Use: Broad phenotype encompassing misuse/problem use; may differ from OUD diagnosis.	PPV	Positive Predictive Value: Proportion of positive predictions that are true positives; clinically, the probability a flagged case is a true case.
QA	Question Answering: Formulating extraction as QA over clinical text.	QuickUMLS	QuickUMLS: Fast, unsupervised UMLS concept matcher commonly used to extract CUIs from clinical text.
RAG	Retrieval-Augmented Generation: Technique that augments LLM generation by first retrieving relevant documents/passages from an external knowledge base.	REMS	Risk Evaluation and Mitigation Strategy: FDA risk program (e.g., for high-risk opioids like TIRF).
RF	Random Forest: Ensemble tree classifier used in several pipelines.	RR	Relative Risk: Association measure used in cohort studies.
SAB	Suggested Aberrant Behavior: Suggested	SBDH	Social and Behavioral Determinants of Health:

	aberrant behavior: narrative cues suggest inappropriate use/drug-seeking without explicit confirmation.		Text-derived behavioral/social risk factors used in downstream analyses.
SD	Standard Deviation: Dispersion summary statistic.	SHRINE	Shared Health Research Information Network: Federated query network/tool referenced in multi-site contexts.
SMART-AI	Substance Misuse Analytics & Real-time Tracking AI: Name of a real-time misuse detection system integrated in EHR workflows.	SNOMED	Systematized Nomenclature of Medicine: Clinical terminology; often used as SNOMED CT.
Snorkel	Snorkel: Weak-supervision framework for building training labels from multiple labeling functions.	SNP	Single-Nucleotide Polymorphism: Genetic variant used in GWAS/PheWAS analyses.
SOAPP	Screener and Opioid Assessment for Patients with Pain: Questionnaire for opioid misuse risk screening.	SPINEL	SPINEL prototype (name): Pharmacovigilance prototype/dashboard name used in an included study.
SVM	Support Vector Machine: Supervised classifier used for note/snippet-level detection.	T5	Text-to-Text Transfer Transformer: Seq2seq transformer model family; used for augmentation.
TF-IDF	Term Frequency–Inverse Document Frequency: Text vectorization baseline feature representation.	TIRF	Transmucosal Immediate-Release Fentanyl: High-risk opioid formulation; referenced in safety and REMS context.
UC	University of California: As used for UC Davis medical center contexts.	UCSD	University of California, San Diego: Institution referenced in O2-Net network list.
UF	University of Florida: Institution referenced in UF Health IDR study.	UK	University of Kentucky: Institution referenced in Lexington, KY study.
UMLS	Unified Medical Language System: NLM metathesaurus; provides CUIs for text concepts.	UW	University of Wisconsin: Institution referenced in UW Health Epic deployment studies.
VA	Veterans Affairs: U.S. integrated health system; many misuse studies are VA-based.	VHA	Veterans Health Administration: Operational arm of VA delivering healthcare.

VINCI	VA Informatics and Computing Infrastructure: VA analytics environment used for large-scale EHR studies.	VUMC	Vanderbilt University Medical Center: Institution referenced in included studies.

Supplementary Table S2. Glossary of key technical concepts used in this review. Explanation of recurring methodological, workflow, and evaluation concepts used throughout the review (e.g., detect-to-triage, human-in-the-loop, weak supervision, external validation, calibration, alert burden). Definitions are tailored to the review's usage and are intended to standardize terminology across studies and sections.

Category	Term	Explanation
Workflow	detect-to-triage	Positioning models to flag/triage high-risk cases for human adjudication rather than autonomous diagnosis. AI acts as a high-sensitivity filter, surfacing candidate cases and specific "note spans" for clinician adjudication, prioritizing human oversight, and appropriate routing
Workflow	human-in-the-loop	Design pattern where clinicians validate/correct model outputs before action; emphasizes safety and accountability.
Evaluation	external validation	Testing a model on data from a different site/time/population than training to assess generalizability.
Evaluation	temporal split	Train/test separation by time to approximate prospective performance and reduce leakage.
Evaluation	calibration	Agreement between predicted probabilities and observed outcomes; critical for thresholded clinical triage.
Evaluation	isotonic calibration	Nonparametric calibration method used to improve

		probability estimates for deployment.
Evaluation	portability	How well a phenotype/model transfers across institutions, EHR systems, or populations.
Method	weak supervision	Using heuristic rules/labeling functions to create training labels when gold labels are scarce (e.g., Snorkel).
Method	labeling functions	Heuristic rules that label data; combined/denoised to train supervised models.
Method	prompting (zero-shot/few-shot)	LLM inference using instructions alone (zero-shot) or a few examples (few-shot) without full fine-tuning.
Method	prompt-based fine-tuning	Training strategy that adapts models using prompts/templates, sometimes with parameter-efficient methods.
Method	data augmentation	Generating additional labeled or weakly labeled text (e.g., paraphrases) to improve robustness.
Method	multimodal EHR modeling	Combining structured events (labs, meds) with note text for prediction.
Method	ontology matching	Mapping note mentions to standard vocabularies (e.g., UMLS, SNOMED) with assertion/negation handling.
NLP task	named entity recognition	Extracting spans/entities (drugs, behaviors, events) from clinical narratives.
NLP task	phenotyping	Defining and detecting clinical conditions/behaviors (e.g., OUD, overdose) from EHR data.

NLP task	entity extraction	General term for pulling structured fields from free text (often NER/QA).
NLP concept	negation detection	Determining whether a mention is negated (“no misuse”) vs affirmed; crucial in clinical text.
Surveillance	under-ascertainment	When billing codes miss cases captured in documentation; motivates text-based surveillance.
Surveillance	ICD-to-text gap	Discrepancy between documentation-derived cases and ICD-coded cases.
Implementation	number needed to evaluate	Workload metric translating alert volume into manual review burden.
Surveillance	pharmacovigilance	Monitoring medication safety signals, including text-mined adverse events and regulatory dashboards.
